# T-cell immunity induced and reshaped by an anti-HPV immuno-oncotherapeutic lentiviral vector

**DOI:** 10.1038/s41541-024-00894-0

**Published:** 2024-06-10

**Authors:** Ingrid Fert, Laëtitia Douguet, Benjamin Vesin, Fanny Moncoq, Amandine Noirat, Pierre Authié, Sylvain Ciret, Fabien Le Chevalier, Catherine Blanc, Yakov Vitrenko, Pierre Charneau, Laleh Majlessi, François Anna

**Affiliations:** 1 Pasteur-TheraVectys Joint Lab, Institut Pasteur, Université de Paris, Virology Department, 28 Rue du Dr. Roux, F-75015 Paris, France; 2 Institut Pasteur, Université Paris Cité, Biomics Technology Platform, F-75015 Paris, France

**Keywords:** Inactivated vaccines, Lymphocyte activation

## Abstract

We recently developed an immuno-oncotherapy against human papillomavirus (HPV)-induced tumors based on a lentiviral vector encoding the Early E6 and E7 oncoproteins of HPV16 and HPV18 genotypes, namely “Lenti-HPV-07”. The robust and long-lasting anti-tumor efficacy of Lenti-HPV-07 is dependent on CD8^+^ T-cell induction and remodeling of the tumor microenvironment. Here, we first established that anti-vector immunity induced by Lenti-HPV-07 prime has no impact on the efficacy of a homologous boost to amplify anti-HPV T-cell immunity. To longitudinally monitor the evolution of the T-cell repertoire generated after the prime, homologous or heterologous boost with Lenti-HPV-07, we tracked T-cell clonotypes by deep sequencing of T-Cell Receptor (TCR) variable β and α chain mRNA, applied to whole peripheral blood cells (PBL) and a T cell population specific of an immunodominant E7_HPV16_ epitope. We observed a hyper-expansion of clonotypes post prime, accompanied by increased frequencies of HPV-07-specific T cells. Additionally, there was a notable diversification of clonotypes post boost in whole PBL, but not in the E7_HPV16_-specific T cells. We then demonstrated that the effector functions of such Lenti-HPV-07-induced T cells synergize with anti-checkpoint inhibitory treatments by systemic administration of anti-TIM3 or anti-NKG2A monoclonal antibodies. While Lenti-HPV-07 is about to enter a Phase I/IIa clinical trial, these results will help better elucidate its mode of action in immunotherapy against established HPV-mediated malignancies.

## Introduction

When become persistent, HPV infections are responsible for almost all cervical, anogenital and many oropharyngeal cancers^1^. The two most abundant HPV genotypes, HPV16 and HPV18, are also the most at risk and are responsible for 71% of cervical cancers^2^. HPV16 and HPV18 have respectively a prevalence of 80% and 3% in oropharyngeal cancers^3^. We have recently developed an anti-HPV16/18 immunotherapy based on a non-integrative lentiviral vector^4,5^, known as “Lenti-HPV-07”, which encodes a polyprotein composed of the detoxified sequences of the Early (E)6 and E7 oncoproteins of HPV16 and HPV18^6^. One of the main advantages of the lentiviral vectors as a vaccine platform is their ability to transduce dendritic cells in vivo. We optimized this feature by using the human β2-microglobulin promoter which is particularly active in activated dendritic cells^7^, to govern the HPV-07 polyantigen expression. The transgenic antigen is thus efficiently and durably expressed by these performant antigen-presenting cells, which effectively induce strong and long-lasting T cell responses at a very low inflammatory response cost^8,9^.

The protective efficacy of Lenti-HPV-07 results from the induction of robust CD8^+^ T cell-based immunity^6^. Lenti-HPV-07 treatment induces a profound remodeling of immune infiltrates and a conversion of the tumor microenvironment to a pro-inflammatory state, which is a highly favorable factor for the success of immuno-oncotherapy^10^. Lenti-HPV-07 therapy is 100% effective in mice with small (≈70 mm^3^), medium (≈200 mm^3^) or large (≈450 mm^3^) tumors, in the standard preclinical model of TC1 cells in C57BL/6 mice. This treatment also completely eradicates lung metastases in 100% of animals, which correlates with the induction of memory resident mucosal CD8^+^ T cells in the lung parenchyma. The immunity induced by Lenti-HPV-07 is long-lasting and completely prevents tumor relapse. We showed that the T cells induced by this vaccine have the required characteristics Programmed cell Death protein (PD)1^+^ and T cell factor-1 (TCF-1)^+^ to be re-invigorated by anti-PD1 treatment. A synergistic anti-tumor effect of anti-PD1 treatment and Lenti-HPV-07 vaccination has been well established^6^. The strong efficacy of Lenti-HPV-07 treatment in the preclinical model led us to set up the preparation of a phase I/IIa clinical trial.

In the preclinical model, a single intramuscular (i.m.) injection of Lenti-HPV-07 is capable of completely eradicating solid tumors in 100% of individuals^6^. However, for clinical use, given the often-weakened immune system of cancer patients, more than one injection may be required to induce a sufficient anti-tumor immunity. In a prime-boost protocol, the Vesicular Stomatitis Virus envelope Glycoprotein (VSV-G), used for lentiviral vector particle pseudotyping, can induce anti-vector humoral and/or cellular immunity that could potentially hinder the efficacy of a homologous boost^11,12^. However, the possible impact of such anti-vector immunity has not yet been assessed in the case of lentiviral vectors. The latter are mainly pseudotyped by VSV-G of two serotypes, Indiana and New Jersey, which are distinguished by their size, i.e., 511 and 517 amino acids, respectively, protein sequences (only 50% identity), post translational modifications and antibodies able to neutralize them^13^. In the present study, by using Lenti-HPV-07 pseudotyped with VSV-G from Indiana (Lenti-HPV-07_ind_) or New Jersey (Lenti-HPV-07_nj_) serotype in prime-boost immunizations, we asked whether: (1) a primary injection of Lenti-HPV-07_ind_ can induce vector-specific adaptive immunity, (2) such immunity could impact the efficacy of a homologous boost, and (3) heterologous boost may have some advantage for T-cell induction.

We then got insight into the magnitude and fine diversity of T-cell responses in mice by longitudinal tracking of clonotypes in mice after Lenti-HPV-07_ind_ prime and after homologous (Lenti-HPV-07_ind_) or heterologous (Lenti-HPV-07_nj_) boost by next-generation sequencing (NGS)-based analysis of TCR Variable (V) β and α domains^14–16^ in peripheral blood cells. A hyper-expansion of clonotypes was correlated with the increased frequencies of HPV-07-specific T cells after prime, and a notable diversification of clonotypes after the boost, underlying a strong remodeling dynamic of the TCR repertoire, which broadens T-cell poly-clonality. However, upon monitoring the specific TCR repertoire associated with the highly immunogenic E7_HPV16_ epitope in H-2b mice (RAHYNIVTF)^17^, we observed that this diversification does not influence clonotypes associated with the immunodominant epitope. This suggests that the diversification primarily affects clonotypes recognizing sub-dominant epitopes.

We finally evaluated the potential of these Lenti-HPV-07-induced T cells to be unleashed by immune checkpoint inhibitor (ICI) treatment, i.e., monoclonal antibodies (mAbs) specific to T-cell immunoglobulin and mucin domain-3 (TIM3)^18^ or NK group 2 member A (NKG2A)^19^, two negative regulatory immune checkpoints expressed by T cells. We previously demonstrated the synergistic effect of Lenti-HPV-07 and anti-PD1 mAb^6^. However, most patients with advanced solid tumors are refractory to this classical ICI therapy^20^, hence our interest in evaluating the therapeutic effect of new ICIs to be combined with Lenti-HPV-07. Interaction of TIM3 with one of its four ligands, galectin-9 (Gal-9)^18^ inhibits cancer immunity by T-cell exhaustion^21^ and it is known that, in the tumor context, TIM3^+^ PD1^+^ T cells are less functional than TIM3^−^ PD1^+^ T cells^22^. NKG2A is an intracytoplasmic tyrosine-based inhibitory motifs (ITIM)-bearing receptor expressed at the surface of 50% of peripheral blood NK cells and 5% of human peripheral blood CD8^+^ T cells. NKG2A is expressed as a heterodimer with CD94 and interacts with non-classical Major Histocompatibility Complex class I (MHC-I) molecules. This interaction inhibits both T and NK effector anti-tumor functions^19^. An anti-NKG2A mAb can be used as ICI, unleashing not only NK, but also CD8^+^ T cells^19^. Our results provided evidence of notable synergistic effect of both anti-TIM3 and anti-NKG2A mAbs on anti-HPV effector T cells induced by Lenti-HPV-07 immunotherapy.

## Results

### No interference of anti-vector immunity with T-cell immunogenicity of Lenti-HPV-07 in a prime-boost regimen

**Fig. 1:**
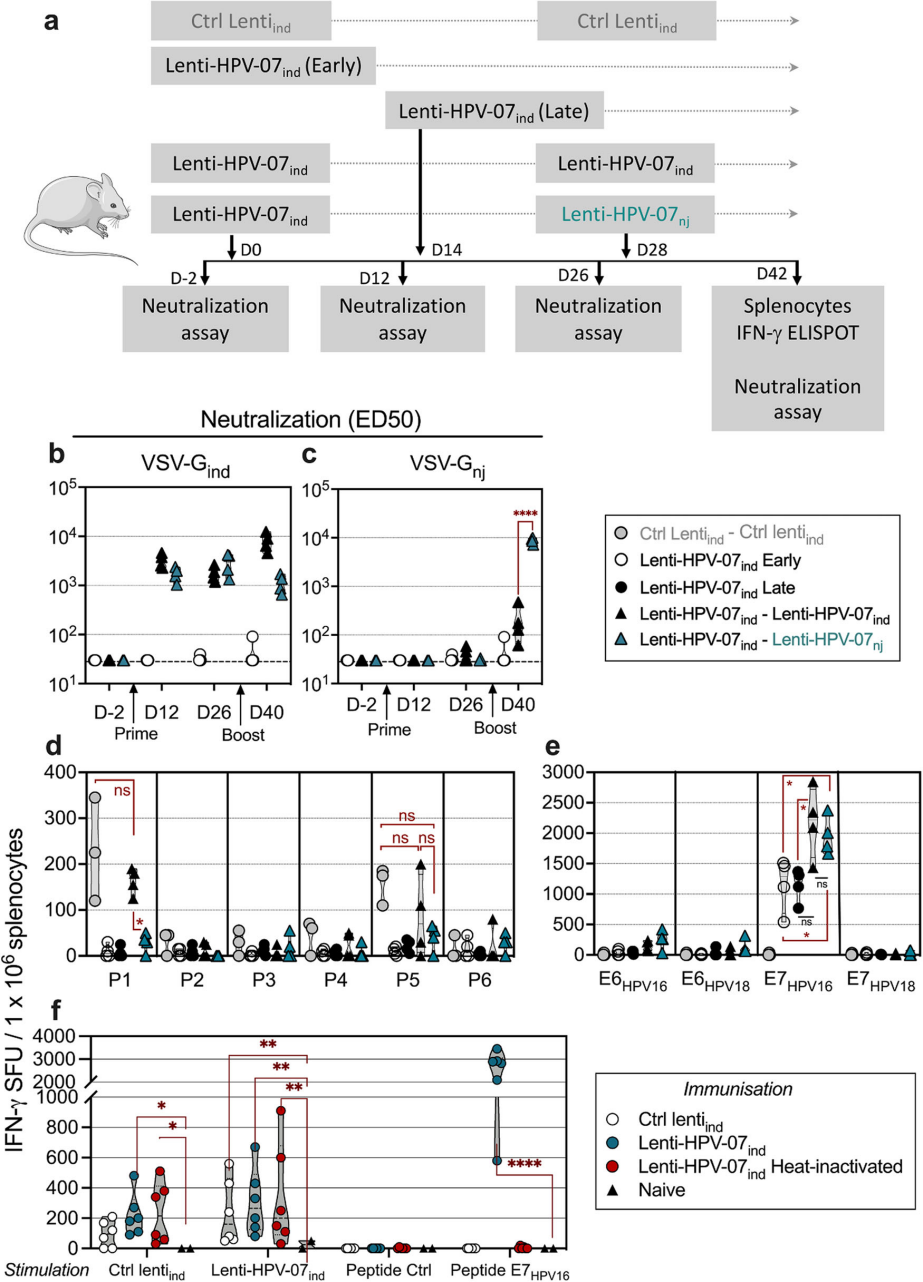
Anti-vector immunity and T-cell immunogenicity of Lenti-HPV-07 in a prime-boost regimen. **a** Timeline of single injection or prime-boost immunization with 1 × 10^9^ TU of Lenti-HPV-07_ind_ or Lenti-HPV-07_nj_, antibody and T-cell assays. **b**, **c** ED50 comparison of anti-VSV-G_ind_ neutralizing antibodies in sera from mice primed with Lenti-HPV-07_ind_ and boosted homologously with Lenti-HPV-07_ind_ or heterologously with Lenti-HPV-07_nj_. ED50 against VSV-G_ind_ (**b**) or against VSV-G_nj_ (**c**) (*n* = 4/group). Statistical significance was determined using a repeated measures (RM) two-way ANOVA (*p < 0.05, ***p < 0.001, ****p < 0.001). IFN-γ ELISPOT responses of T splenocytes from the same individual mice were studied at the indicated time points after in vitro stimulation with (**d**) six peptide pools spanning the full sequence of VSV-G_ind_, or (**e**) four peptide pools spanning the sequence of detoxified E6_HPV16_, E7_HPV16_, E6_HPV18_ or E7_HPV18_, as encoded by Lenti-HPV-07. **f** IFN-γresponses of T splenocytes of mice assessed at D14 after a mono-injection with 1 × 10^9^ TU of Ctrl lenti_ind,_ Lenti-HPV-07_ind_ or Lenti-HPV-07_ind_ heat-inactivated at 70 °C during 1 h (*n* = 6). Naive mice served as control (*n* = 2). Stimulation was performed using either the indicated lentiviral vectors or peptides both at 4 µg/ml. SFU spot forming unit. Statistical significance was determined by Mann–Whitney *t*-test (ns not significant, *p < 0.05, **p < 0.01, ****p < 0.0001).

Since anti-vector immunity induced by prime may reduce the efficacy of a homologous boost^11,12^, we studied the possible impact of anti-VSV-G immunity on the T-cell immunogenicity of Lenti-HPV-07 therapeutic vaccine candidate in a prime-boost regimen. C57BL/6 mice were immunized i.m. at day (D) 0 with 1 × 10^9^ TU of Lenti-HPV-07 pseudotyped with VSV-G from Indiana serotype (Lenti-HPV-07_ind_). Then, groups of primed mice were left unboosted or were boosted i.m. at D28 with 1 × 10^9^ TU of homologous Lenti-HPV-07_ind_ or heterologous Lenti-HPV-07 pseudotyped with VSV-G from New Jersey serotype (Lenti-HPV-07_nj_) (*n* = 4/group) (Fig. 1a). A group of mice received a single injection of Lenti-HPV-07_ind_ at D14 to compare the persistence of T-cell response levels. Another group was primed and boosted with the same amounts of an empty lentiviral vector (“Ctrl Lenti_ind_”).

We first assessed the anti-VSV-G_ind/nj_ neutralizing antibodies. Sera were collected pre prime (D-2), post prime (D12 and D26), and post boost (D40). The effective dose 50 (ED50), inhibiting the vector entry into permissive HEK293T cells, was determined in the serum serial dilutions (Fig. 1b, c). Anti-VSV-G_ind_ neutralizing activity was measurable at D12 post Lenti-HPV-07_ind_ prime and increased significantly after a homologous boost. In Lenti-HPV-07_ind_-primed mice, heterologously boosted with Lenti-HPV-07_nj_, the amounts of anti-VSV-G_ind_ neutralizing antibodies were significantly weaker (Fig. 1b). Therefore, a heterologous boost avoids generation of vector neutralizing antibodies. Weak amounts of anti-VSV-G_nj_ neutralizing antibodies were detectable in Lenti-HPV-07_ind_-primed mice, homologously boosted with Lenti-HPV-07_ind_, whereas such antibodies were readily detectable in their counterparts heterologously boosted with Lenti-HPV-07_nj_ (Fig. 1c).

In the same mice, analysis of anti-VSV-G-specific IFN-γ T splenocyte responses, following in vitro stimulation with pools of VSV-G_ind_-derived overlapping 15-mers, showed near-to-background levels of responses after the Lenti-HPV-07_ind_ prime (Fig. 1d). T-cell responses against the VSV-G_ind_ peptide pools #1 and #5, containing the predicted H-2D^b^/K^b^ epitopes (Supplementary Table 1), were detected in mice homologously primed and boosted with Ctrl Lenti_ind_ or Lenti-HPV-07_ind_. Such T-cell responses were detected in a much lesser extent in mice primed with Lenti-HPV-07_ind_ and heterologously boosted with Lenti-HPV-07_nj_. Sequence alignment and epitope prediction showed that the VSV-G_ind_ predicted epitopes, contained in these peptide pools have only 5/9, 1/8, 3/8 and 6/8 a.a. sequence identities with VSV-G_nj_ (Supplementary Fig. 1 and Supplementary Table 1), which can explain the reason why T-cell responses to these epitopes were not boosted with Lenti-HPV-07_nj_.

Parallel study of anti-HPV-07 IFN-γ T splenocyte responses in the same mice (Fig. 1e), showed a notable response against E7_HPV16_ and weaker responses against E6_HPV16_ and E6_HPV18_, in accordance with previous results^6^. Mice immunized at D0 or D14 with a single injection of Lenti-HPV-07_ind_ had comparable responses to E7_HPV16_, showing the persistence of the lentiviral vector-induced responses^7,8^. Importantly, significant and comparable increases in the frequencies of T cells against E7_HPV16_ were recorded after boost, regardless of the serotype of VSV-G used in pseudotyping of Lenti-HPV-07 used for boost (Fig. 1e). The presence of an anti-VSV response (Fig. 1d) points out a detectable anti-vector T immunogenicity. Moreover, the vector production process entails the presence of cellular residues, which may persist despite multiple purification steps, potentially eliciting another immune response unrelated to the encoded HPV antigens. The overall immunogenic potential of our vectors was assessed in mice at D14 after a mono-injection of Ctrl Lenti_ind_, Lenti-HPV-07_ind_ or heat-inactivated Lenti-HPV-07_ind_ (*n* = 6). T splenocyte response was detected using IFN-γ ELISPOT (Fig. 1f) or by a T-cell activation assay based on upregulation of the early activation marker CD69 by CD8^+^ T cells co-cultured with bone-marrow-derived dendritic cells incubated with the vector preparations used for vaccination (Supplementary Fig. 2). In both Ctrl Lenti_ind_ and Lenti-HPV-07_ind_ groups, anti-vector T-cell responses, which may include anti-VSV-G responses, were detected. Comparable responses detected in mice vaccinated with heat-inactivated Lenti-HPV-07_ind_, implies that T cell activation against the vector backbone is not dependent on vector functionality. It is noteworthy that the vector samples elicited anti-vector T-cell responses which were much less intense than those induced against the transgenic HPV antigens (Fig. 1d–f).

In parallel, in the mice vaccinated with heat-inactivated Lenti-HPV-07_ind_, no discernible response was detected against the transgenic HPV antigens (Fig. 1f and Supplementary Figs. 2 and 3). This result showed that residual proteins originated from transgene expression during the vector production and possibly still present in the vector samples do not induce detectable specific T-cell responses. Therefore, induction of specific T-cell responses by these vectors relies only on their in vivo transduction ability. Altogether, a homologous or heterologous boost with Lenti-HPV-07_ind_ or Lenti-HPV-07_nj_ increased the intensity of the specific anti-HPV T-cell effectors without any sizeable impact of the adaptive immunity against the vector backbone.

### Monitoring the TCR repertoire throughout a Lenti-HPV-07 prime-boost immunization regimen

**Fig. 2:**
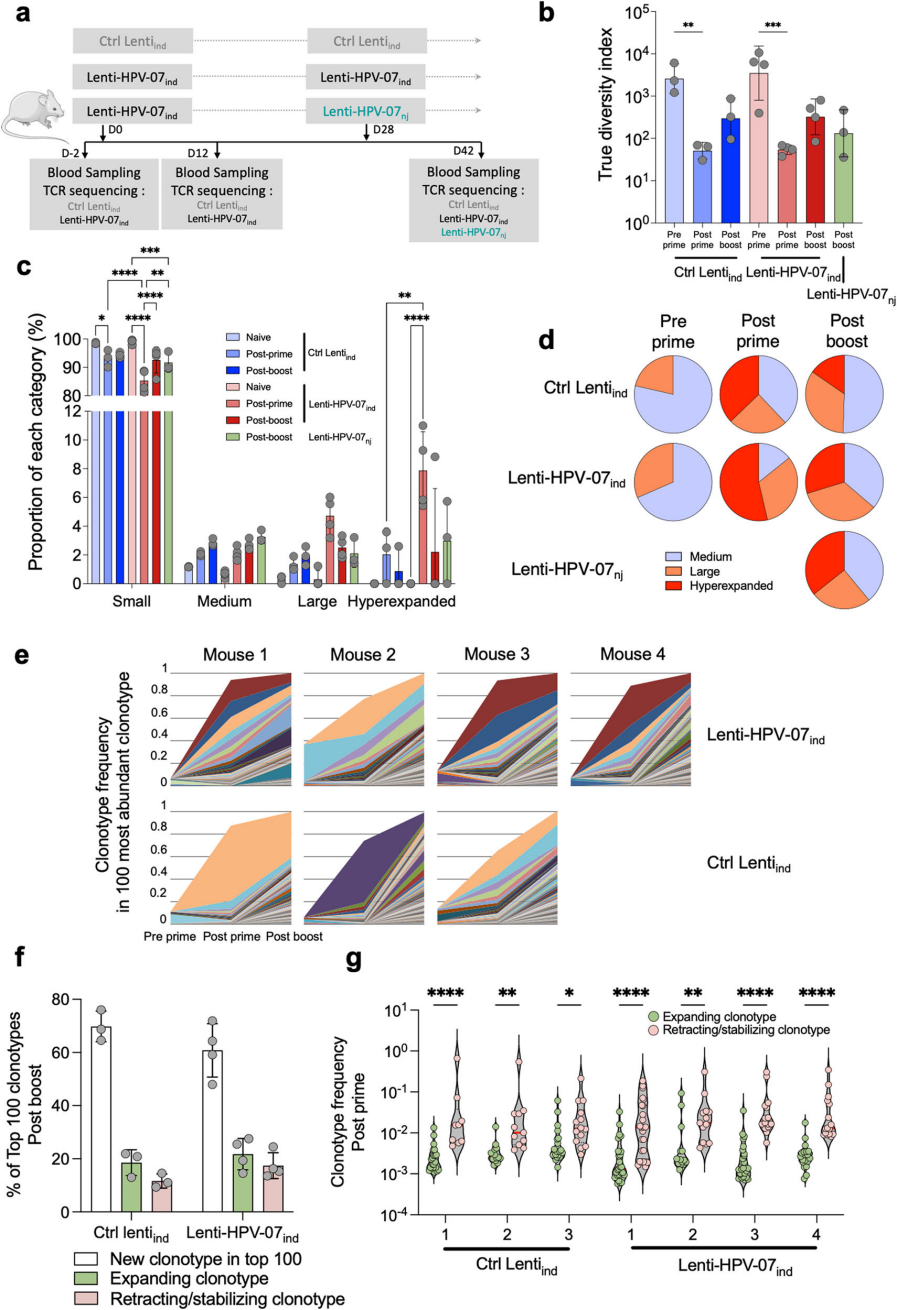
Dynamic evolution of TCRβ repertoire throughout a Lenti-HPV-07 prime-boost immunization regimen. **a** Timeline of prime-boost immunizations with 1 × 10^9^ TU of Lenti-HPV-07_ind_, Lenti-HPV-07_nj_ or Ctrl Lenti_ind_ and TCRβ sequencing, performed on RNA extracted from peripheral blood cells. **b** Estimation of the TCRβ repertoire diversity using “true diversity” index. **c** Relative abundance of clonotypes, defined as their frequencies (X), compared to the total number of reads, which allows to divide the clonotypes into Small (0 < X ≤ 10^–4^), Medium (10^–4^ < X ≤ 10^–3^), Large (10^–3^ < X ≤ 10^–2^) and hyperexpanded (10^–2^ < X ≤ 10^0^) categories. Relative abundance is the sum of the frequencies of all clonotypes from a given category. **d** Pie chart representing average proportions (mean) of Medium, Large and hyperexpanded categories in each group. **e** Follow up of the frequencies for the top 100 most abundant clonotypes, as defined at the post boost timepoint. **f** Classification of the top 100 most abundant clonotypes detected post boost, according to their expanding or contracting evolution since prime. New post boost clonotypes were defined from the comparison with top 100 post prime. Expanding or retracting/stabilizing clonotypes were defined as those having increasing or decreasing frequencies post boost respectively. **g** Frequencies of expanding or contracting clonotypes. Statistical significance was determined using a one-way ANOVA in (**b**) and, two-way ANOVA (*p < 0.05, **p < 0.01, ***p < 0.001, ****p < 0.001) in (**c**, **f**). Error bars on the histograms represent standard deviation.

To investigate the magnitude and fine diversity of T-cell responses induced by Lenti-HPV-07_ind_ immunization, we performed deep sequencing of TCR Variable -β and -α regions in peripheral blood cells throughout a prime-boost immunization regimen. C57BL/6 mice were primed i.m. with Lenti-HPV-07_ind_ (*n* = 3 or 4/group) on D0 and then boosted with Lenti-HPV-07_ind_ or Lenti-HPV-07_nj_ on D28 (Fig. 2a). Control mice were primed and boosted with Ctrl Lenti_ind_ (*n* = 3). Total RNA was extracted from peripheral blood cells on D-2 (pre prime), D12 (post prime), and D40 (12 days post boost). Libraries corresponding to V(D)J variable regions of TCR transcripts were generated by a procedure leveraging 5’ rapid amplification of cDNA (5’ RACE) and sequenced by NGS^14–16^. Paired reads were aligned yielding about 5 × 10^5^ usable sequences per sample. Vaccination-induced “clonotypes” were monitored by the identification of unique rearrangements of Variable (V)β and Joining (J)β segments, within the third Complementarity-Determining Region (CDR3). The diversity of TCRβ repertoire was described by: (1) “richness” which indicates the total numbers of distinct clonotypes present per sample, and (2) “abundance” which evaluates the clonotype expansion^15^. However, clonotype counts could not be considered as an exhaustive measurement of the complete TCR repertoire. In addition to the richness and abundance, we used Shannon entropy to estimate the “true diversity” including “unseen” clonotypes linked to sampling limitation^23^.

Priming with both Lenti-HPV-07_ind_ or Ctrl Lenti_ind_ compressed the TCRβ (Fig. 2b) and TCRα repertoires manifested as a significative 1-log reduction of the true diversity index recorded at D12, compared to the pre prime baseline (Supplementary Fig. 2). We argue that this reduction reflects the selection/amplification of specialized Vβ-Jβ rearrangements and is followed post boost by a non-statistically significant increase. This, in turn, could be associated with a boost-mediated diversification of the vector/vaccine-specific clonotypes (Fig. 2b and Supplementary Fig. 4). Since the same repertoire variations were recorded in Lenti-HPV-07_ind_ and Ctrl Lenti_ind_ samples, at least part of them seems to be related to anti-vector T-cell responses, independent of those specific to the transgenic HPV-07 antigen, in accordance with the anti-vector T-cell responses detected in the Ctrl Lenti_ind_-injected mice (Fig. 1d, f).

We analyzed the clonotype abundance, defined by the frequency (X) as the ratio of clonotype specific read counts relative to the total number of reads. Then we range clonotypes into the following frequency categories: small (0 < X ≤ 10^−4^), medium (10^–4^ < X ≤ 10^–3^), large (10^–3^ < X ≤ 10^–2^) or hyperexpanded (10^–2^ < X ≤ 10^0^) categories. The post prime compression of the TCRβ repertoire (Fig. 2b) was accompanied by an increase in the relative abundance of clonotypes in the medium, large or hyperexpanded categories (Fig. 2c). While the post prime TCRβ repertoire was characterized by increased proportions of hyperexpanded clonotypes, the post boost TCRβ repertoire showed similar proportions of clonotypes in the large and hyperexpanded categories (Fig. 2c, d). Similar results were observed for TCRα repertoire (Supplementary Fig. 2). The proportion of the large category clonotypes has a tendence to grow, and a statistically significative increase was observed for hyperexpanded clonotypes (2.0 ± 1.8% in Ctrl Lenti_ind_ vs. 7.9 ± 2.7% in Lenti-HPV-07_ind_) post prime group which is likely to be associated with antigen-specific T-cell responses (Fig. 2c, d). Longitudinal tracking of the 100 most abundant post boost clonotypes showed a post prime and post boost increase and/or maintenance of frequency variation since the pre prime timepoint (Fig. 2e). Part of the clonotypes have an increased frequency, hallmark of clonal expansion, as early as post prime, while some others need the booster injection to expand. Half of those detectable at both post prime and post boost timepoints, displayed increasing frequencies, whereas the other half had decreasing frequencies, possibly due to a partial T-cell contraction (Fig. 2f). Interestingly, the expanding clonotypes displayed about 1-log lower post prime frequencies than the contracting clonotypes (Fig. 2g). Such a distribution, observed in both Ctrl Lenti_ind_ and Lenti-HPV-07_ind_ groups, may indicate a post boost retraction/stabilization of the initial clonotypes, possibly specific to immunodominant epitopes, followed by the expansion of rare, subdominant epitope-specific clonotypes emerging post boost to diversify the T-cell repertoire.

**Fig. 3:**
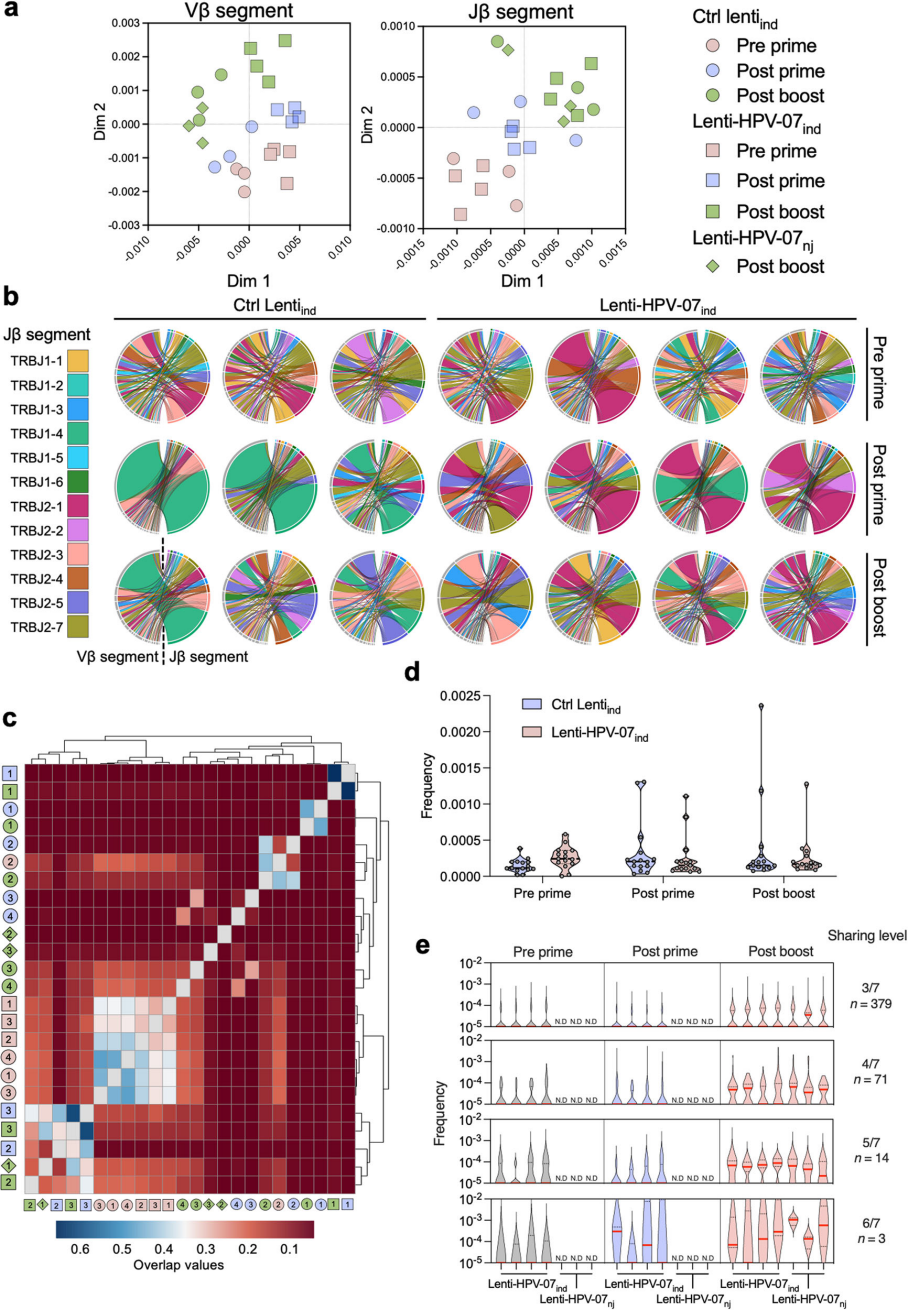
Vβ-Jβ rearrangements detected in Lenti-HPV-07-immunized mice. Samples are those detailed in Fig. 2a. **a** Jβ- and Vβ-gene segment usage in all clonotypes from each sample. For each clonotype, the frequency of Jβ and Vβ usage was determined as detailed in Supplementary Figs. 2 and 3. Jβ and Vβ segment repertoire usage was compared among all mice at all timepoints according to a Jensen-Shannon Divergence model as detailed in Supplementary Figs. 2 and 3 and treated by Multidimensional Scaling (MDS) to represent repertoire usage gene proximity on a 2-dimension plot. **b** Chord diagrams representing the Jβ and Vβ association of in the top 100 most abundant clonotypes, as defined post boost. Chords are colored according to the Jβ-gene segment. **c** Heatmap repertoire overlap, showing Jβ and Vβ segment usage in association to CDR3 amino acid sequence of each clonotype, using Morisita’s overlap index. Ctrl Lenti (squares) and Lenti-HPV-07 (circles) groups, pre prime (pink), post prime (blue) and post boost (green) timepoints, and mouse numbers are indicated inside the symbols. Cluster dendrograms indicate the unweighted hierarchical proximities between samples. **d** Frequencies of the 15 clonotypes shared by all samples at each time point. **e** Frequencies of clonotypes which exclusively emerged in the Lenti-HPV-07_ind/nj_-injected mice at each time point and their 3-to-6 sharing levels. Numbers of clonotypes per category are indicated on the right. Median of each violin box is indicated in red.

Vβ- and Jβ-segment usage, an indicator of the TCR repertoire polarization toward antigen recognition, was also studied and mutually compared in all mice at all time points, according to a Jensen-Shannon Divergence model (Supplementary Figs. 5 and 6). The data were also treated by Multidimensional Scaling (MDS) to visualize the repertoire usage proximity on a 2-dimension plot (Fig. 3a). For both Vβ and Jβ segments, the repertoire usage clustered in a same manner after the injection of Ctrl Lenti or Lenti-HPV-07_ind_. Importantly, while no significative shift was observed for Vβ- and Jβ-segment usage in Ctrl Lenti_ind_-immunized mice, Lenti-HPV-07 immunization clearly favored the use of TRBJ2.1 and TRBJ2.7 in all mice (Supplementary Fig. 6), which may be associated with HPV-07 antigen-specific clones. The preferentially used TRBJ2.1 and TRBJ2.7 segments were associated with diverse Vβ segments with a preference for TRBV17 (Fig. 3b) in a few largely expanded clonotypes in Lenti-HPV-07-immunized mice. No significant Vα- and Jα-segment usage was observed in the immunized mice, yet it has to be mentioned that the higher numbers of Vα and Jα gene segments^24,25^ may hinder the detection of TCRα repertoire polarization (Supplementary Fig. 7).

In addition to the Vβ and Jβ segment usage, the CDR3 sequence was analyzed using Morisita’s overlap index to calculate the amino acid sequence proximity throughout the entire TCRβ repertoire. The results are represented as a hierarchical clustering heatmap (Fig. 3c). Six of 7 pre prime samples clustered together, suggesting that the syngeneic mice may share a common TCRβ repertoire. Two of the 3 Ctrl Lenti_ind_ samples clustered together post prime and post boost. Lenti-HPV-07_ind_ samples clustered only longitudinally for each mouse without similarities within the group. The same was observed for TCRα repertoire (Supplementary Fig. 7), despite the absence of D segment drastically limits the potential diversity of TCRα chain. To assess the vaccination effect on irrelevant clonotypes, 15 shared clonotypes were identified in all mice at all timepoints, including the pre prime samples (Fig. 3d). These clonotypes, unrelated to immunization, had neglectable variations post prime and post boost in both Ctrl Lenti_ind_ and Lenti-HPV-07_ind/nj_ groups, suggesting that vaccination has no general bystander effect on an irrelevant TCR repertoire.

Despite the poorly overlap of the post vaccination repertoires, the TCR repertoire specialization toward a defined antigen could select clonotypes sharing the same TCRβ sequences. In total, 467 clonotypes were identified post boost which were shared by at least 3 of 7 Lenti-HPV-07_ind/nj_-immunized mice, but not with Ctrl-Lenti_ind_-injected mice. We monitored the evolution of the frequencies of these clonotypes throughout the experiment classifying them from level 3 to 6 according to the number of Lenti-HPV-07_ind/nj_-injected mice which share them (Fig. 3e). For all these levels, clonotype expansion could be visualized only after the boost, pointing to a dependence of the clonal expansion on Lenti-HPV-07_ind/nj_ injection. Within levels 3, 4 and 5, no differences in the frequencies were observed, and the shared clonotypes were mainly of small or medium abundance. Interestingly, most of these clonotypes were not detectable post prime. This observation was in accordance with the potential of the boost to induce an expansion of clonotypes of the small abundance, as observed among the top 100 most abundant clonotypes (Fig. 3e). Within level 6, the most shared clonotypes were mainly of large abundance in all mice and had already expanded post prime. Interestingly, 2 of 3 such CDR3 sequences had been reported in a large-scale public study^26^, explaining the high level of sharing observed here.

### Evolution of T clonotypes exclusively specific to HPV antigens

**Fig. 4:**
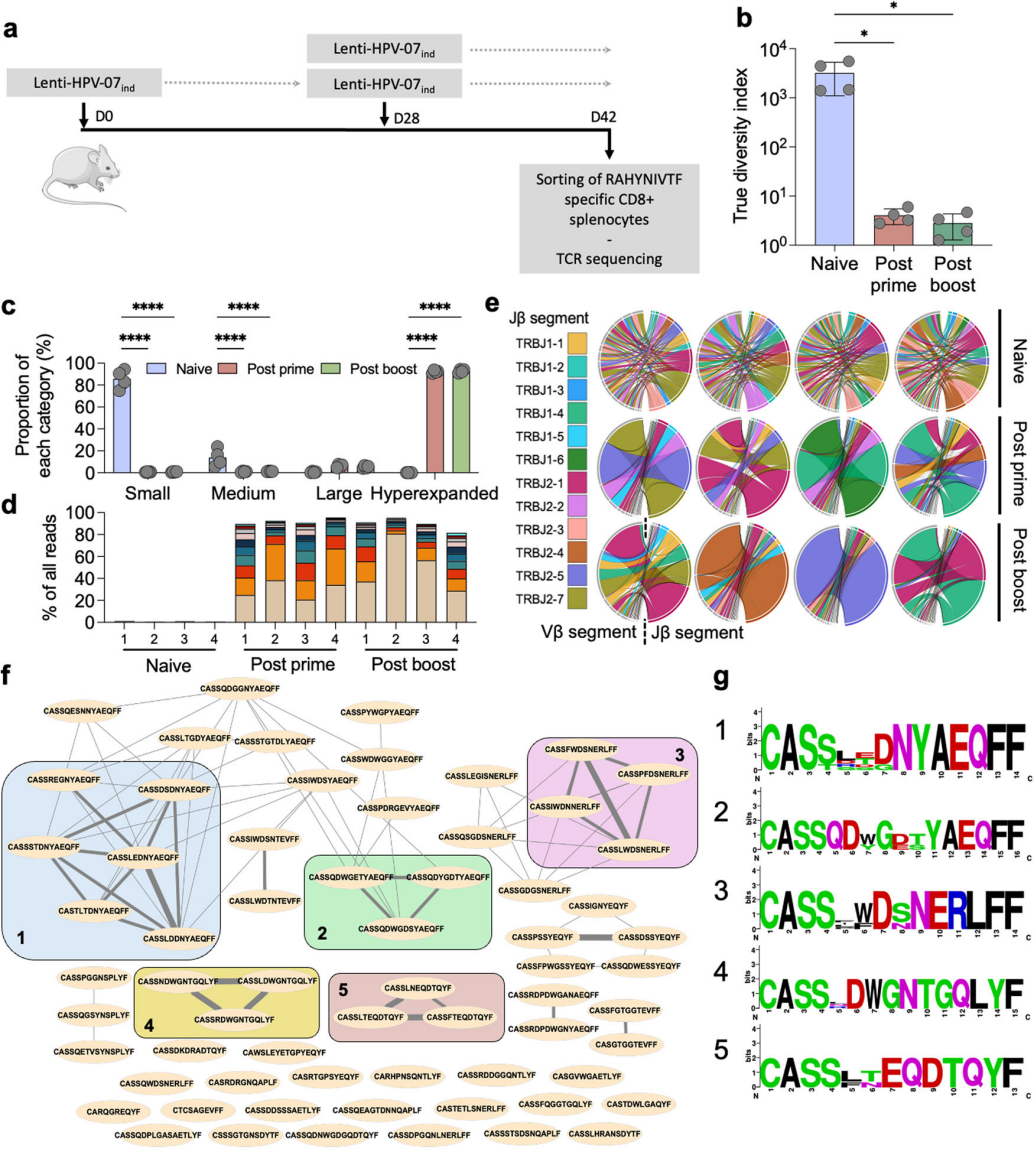
Evolution of the TCRβ repertoire of the clonotypes specific to an immunodominant E7_HPV16_ epitope after a single injection or a prime-boost Lenti-HPV-07_ind_ vaccination. **a** Timeline of a mono-injection or prime-boost (i.m.) with 1 × 10^9^ TU of Lenti-HPV-07_ind_. TCRβ sequencing was performed on bulk RNA extracted from CD8^+^ T splenocytes sorted by use of a PE-conjugated H-2D^b^ (RAHYNIVTF) dextramer (Supplementary Table 2 and Fig. 8). **b** Estimation of the TCRβ repertoire diversity using “true diversity” index. **c** Relative abundance of clonotypes, defined as their frequencies (X), compared to the total number of reads, which allows to divide the clonotypes into Small (0 < X ≤ 10^–4^), Medium (10^–4^ < X ≤ 10^–3^), Large (10^–3^ < X ≤ 10^–2^) and hyperexpanded (10^–2^ < X ≤ 10^0^) categories. Relative abundance is the sum of the frequencies of all clonotypes from a given category. **d** percentage of all reads of the top 10 most abundant clonotypes for each mouse. Each color corresponds to distinct clonotypes from the least abundant (top) to the most abundant (bottom). **e** Chord diagrams representing the Jβ and Vβ association of in the top 100 most abundant clonotypes. Chords are colored according to the Jβ-gene segment. **f** Network representing CDR3 sequence proximity between top 10 most abundant clonotypes of all post prime and post boost mice (*n* = 65 CDR3 sequences). An edge between CDR3 sequences was added according to their LD. LD = 1, large line; LD = 2, medium line; LD = 3, thin line. Groups represent a cluster of CDR3 sequence with an LD < 3. **g** Amino acids logo for each group of related CDR3 sequences. Statistical significance was determined using a one-way ANOVA in (**b**) and, two-way ANOVA (*p < 0.05, ****p < 0.001) in (**c**). Error bars on the histograms represent standard deviation.

To study the evolution of clonotypes exclusively specific to HPV antigens, C57BL/6 mice (*n* = 4/group) were immunized with Lenti-HPV-07 by a single injection or following a prime-boost regimen. CD8^+^ T splenocytes specific for the immunodominant E7_HPV16_ epitope were cytometrically sorted by use of a PE-conjugated H-2D^b^ (RAHYNIVTF) dextramer (Fig. 4a) and their TCRβ repertoire was analyzed by RNAseq. In contrast to the results obtained for the global TCRβ repertoire, that of this epitope-specific CD8^+^ T subset showed no increase in diversity after the boost injection (Fig. 4b). This result suggests that the observed increase in the post-boost global diversity may be attributed to the expansion of clonotypes recognizing sub-dominant epitopes rather than immunodominant ones concordant with previous observation on post boost retraction/stabilization of post prime most abundant clonotypes (Fig. 2g). More than 80% of these clonotypes were hyperexpanded (Fig. 4c), with the top 2 most frequent clonotypes representing >40% of the specific TCRβ repertoire, while the top 10 clonotypes collectively accounted for >80% (Fig. 4d). The biased usage of TRBJ2.1 and TRBV17 but not TRBJ2-7 segments from Vβ and Jβ, previously observed in global repertoire analysis, was found again for the CD8^+^ T cell subset specific to the immunodominant RAHYNIVTF epitope. A pairing bias was also observed between the TRBJ2.1 and TRBV17 chains together (Supplementary Fig. 8) in this specialized TCRβ repertoire of the Lenti-HPV-07 vaccinated mice, compared to that of naive mice. This observation suggests that this combination is favored to generate a TCRβ chain specific to the RAHYNIVTF epitope. Finally, to investigate the similarities among the CDR3 sequences of the top 10 clonotypes in each vaccinated mouse, their peptide sequences were isolated and the Levenshtein distance (LD) between them was calculated^27^. A representation connecting the sequences according to LD was then generated, and 5 clusters of at least 3 sequences with an LD of 2 or less were identified (Fig. 4f). Logo sequence analysis showed biases in the composition of CDR3 sequences (Fig. 4g). The 1-4 N-ter position contained the CASS amino acid consensus sequence, followed by a preferably negatively charged amino acid at the position 6 or 7, mainly aspartate. This residue is mostly surrounded by a tryptophan and/or glycine in position 6-7-8.

These results globally highlight the initiation and evolution of clonotypes mobilized by Lenti-HPV-07 immunization, especially after a boost, which allows the expansion of subdominant clonotypes and favorizes the diversification of antigen-specific TCR repertoire. However, monitoring the E7_HPV16_ specific T cells indicated that a boost immunization did not significantly alter the clonotype diversity within a T subset specific to an immunodominant epitope. It must be mentioned that this strategy has inherent limitations, as demonstrated here, due to the lack of antigen specificity when evaluating the efficacy of lentiviral vectors, and to a broader extent, other immunogenic carriers or viral vectors that can trigger non-specific responses.

### Lenti-HPV-07-induced T cells act synergistically with anti-TIM3 or anti-NKG2A anti-check point immunotherapy

Having thoroughly characterized the clonotypes that take part in the T-cell response induced by Lenti-HPV-07 vaccination, we then examined the anti-tumor function of these T cells and the possibility of disinhibiting their anti-tumor effector functions by ICI treatments.

**Fig. 5:**
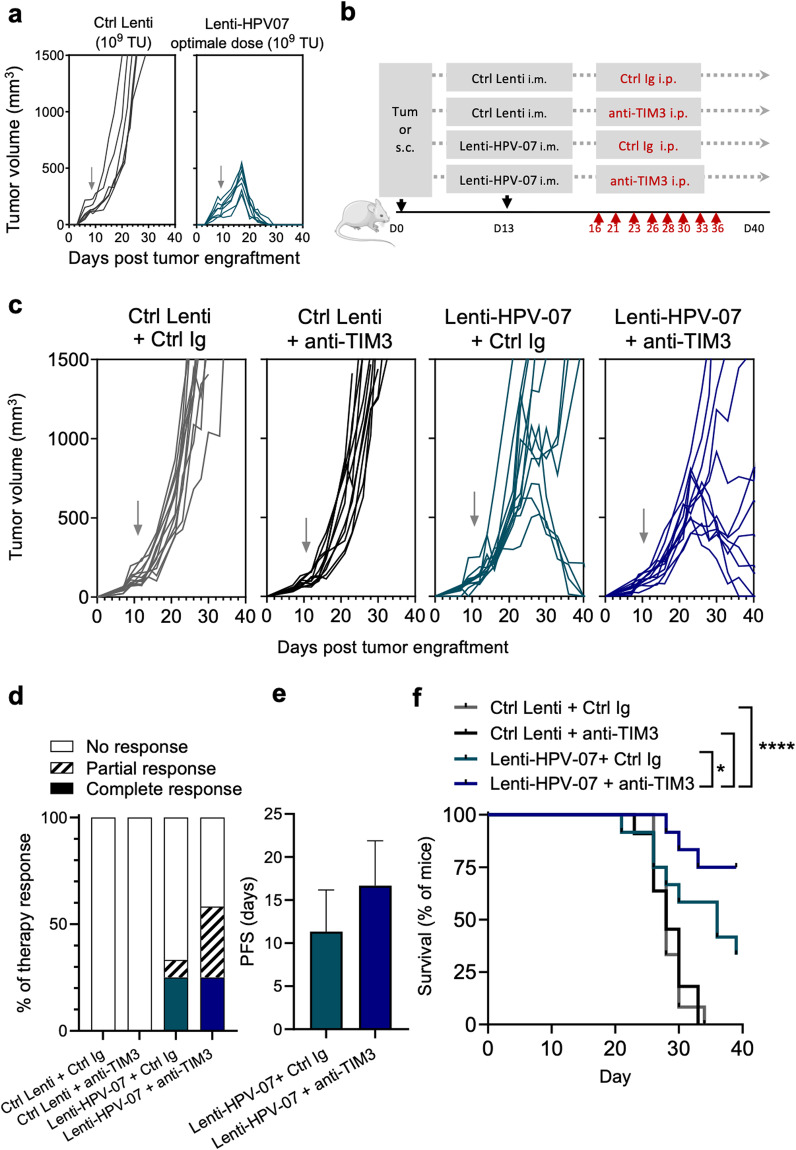
Synergistic effect of Lenti-HPV-07 and anti-TIM3 mAb in TC1 tumor regression. **a** C57BL/6 mice were s.c. flank-engrafted with 1 × 10^6^ of TC1 cells. When the tumor volume reached an average of 130–140 mm^3^, mice were vaccinated with the optimal dose of 1 × 10^9^ TU/mouse of Lenti-HPV-07 (*n* = 7) or a Ctrl Lenti (*n* = 6). Tumor growth was monitored with electronic caliper. **b** Timeline of tumor engraftment and combinatory treatment with Lenti-HPV-07 and anti-TIM3 mAb. C57BL/6 mice (*n* = 11–12/group) were s.c. flank-engrafted with 1 × 10^6^ of TC1 cells. When the tumor volume reached an average of 130 mm^3^ (day 13), mice were randomized and vaccinated with the suboptimal dose of 1 × 10^8^ TU/mouse of Lenti-HPV-07 or a Ctrl Lenti. Mice were then treated with anti-TIM3 mAb (clone RMT3–23, Bioxcell) or Ig control (clone 2A3, Bioxcell) 2 or 3 times a week. A total of 8 injections was given from day 16 to day 36 (200 µg/injection). **c** Spaghetti plots of tumor growth. **d** Therapy response rates according to RECIST criteria as follows. Complete response is defined by the complete eradication of the tumor observed during the time of the experiment. Partial response is defined by at least two consecutive decreasing measures during the treatment. Progression-free survival (PFS) is defined in all mice to reflect the duration of the response; from the day that tumor reached their maximum size until the day that tumor exceeded the maximum size again. **e** PFS of mice bearing TC1 tumors and treated with Lenti-HPV-07 and TIM3 blocking antibody. **f** Survival curves of the animals shown in (**c**). Statistical significance was determined using a Log-rank Mantel–Cox test, *p = 0.0465, ****p < 0.0001) in (**f**). Mice were sacrificed when the size of the tumors reached 1500 mm^3^, in accordance with the defined humane endpoints. Error bars on the histograms represent standard deviation.

As mentioned above, a single i.m. injection of the optimal dose of 1 × 10^9^ TU/mouse of Lenti-HPV-07 is sufficient to fully eradicate E6_HPV16_- and E7_HPV16_-expressing TC1 tumors in 100% of C57BL/6 mice, bearing small, medium or large tumors^6^. This observation was reproduced and confirmed here on mice bearing TC1 tumors with an average size of 130–140 mm^3^ (Fig. 5a). The anti-tumor action of Lenti-HPV-07-induced T cells can be enhanced by combining this vaccination with anti-PD1 ICI treatment^6^. Here, we further evaluated the efficacy of a therapeutic combination of Lenti-HPV-07 with mAbs against TIM3 or NKG2A. It was previously shown that therapeutic HPV vaccination induced TIM3, NKG2A and PD-1 expression by more than 80% of CD8^+^ tumor infiltrating T cells^28^.

Since the optimal dose of 1 × 10^9^ TU/mouse Lenti-HPV-07 completely eradicates TC1 tumors in 100% of animals, we used the suboptimal dose of 1 × 10^8^ TU/mouse Lenti-HPV-07 that partially cured TC1 tumor-bearing mice. We first explored the potential of anti-TIM3 (clone RMT3–23) mAb treatment started 3 days after a single injection of Lenti-HPV-07. Control animals received 1 × 10^8^ TU of Lenti-HPV-07 and a control Ig (Ctrl Ig). Other groups received Ctrl Lenti + Ctrl Ig or Ctrl Lenti + anti-TIM3 mAb (Fig. 5b). In the Ctrl Lenti + Ctrl Ig and Ctrl Lenti + anti-TIM3 mAb groups, no effect on tumor regression was recorded. Therefore, anti-TIM3 mAb treatment alone has no effect on TC1 tumor growth (Fig. 5c). In the Lenti-HPV-07 + Ctrl Ig group only 4 of 12 mice achieved tumor regression versus 8 of 12 which were unable to control tumor growth (Fig. 5c). Conversely, in the Lenti-HPV-07 + anti-TIM3 mAb group, 8 of 12 mice achieved tumor regression while the other 4 were unable to control tumor growth. Strikingly, the combinatory treatment increased the response: 60% of mice treated with Lenti-HPV-07 + anti-TIM3 mAb displayed a complete or partial regression response, while Lenti-HPV-07 alone caused the response only in 30% of mice (Fig. 5d). The progression-free survival (PFS) time was longer in mice that received the combination of Lenti-HPV-07 and anti-TIM3 mAb compared to the mice treated with the Lenti-HPV-07 + Ctrl Ig, even though the difference did not reach statistical significance (Fig. 5e). Accordingly, the survival significantly increased in the group with combinatory treatment (Fig. 5f). Because anti-TIM3 mAb alone had no impact on TC1 tumor growth and Lenti-HPV-07 + anti-TIM3 mAb was more efficient than Lenti-HPV-07 + Ctrl Ig, the effect of combination therapy appears to be rather synergistic than additive.

**Fig. 6:**
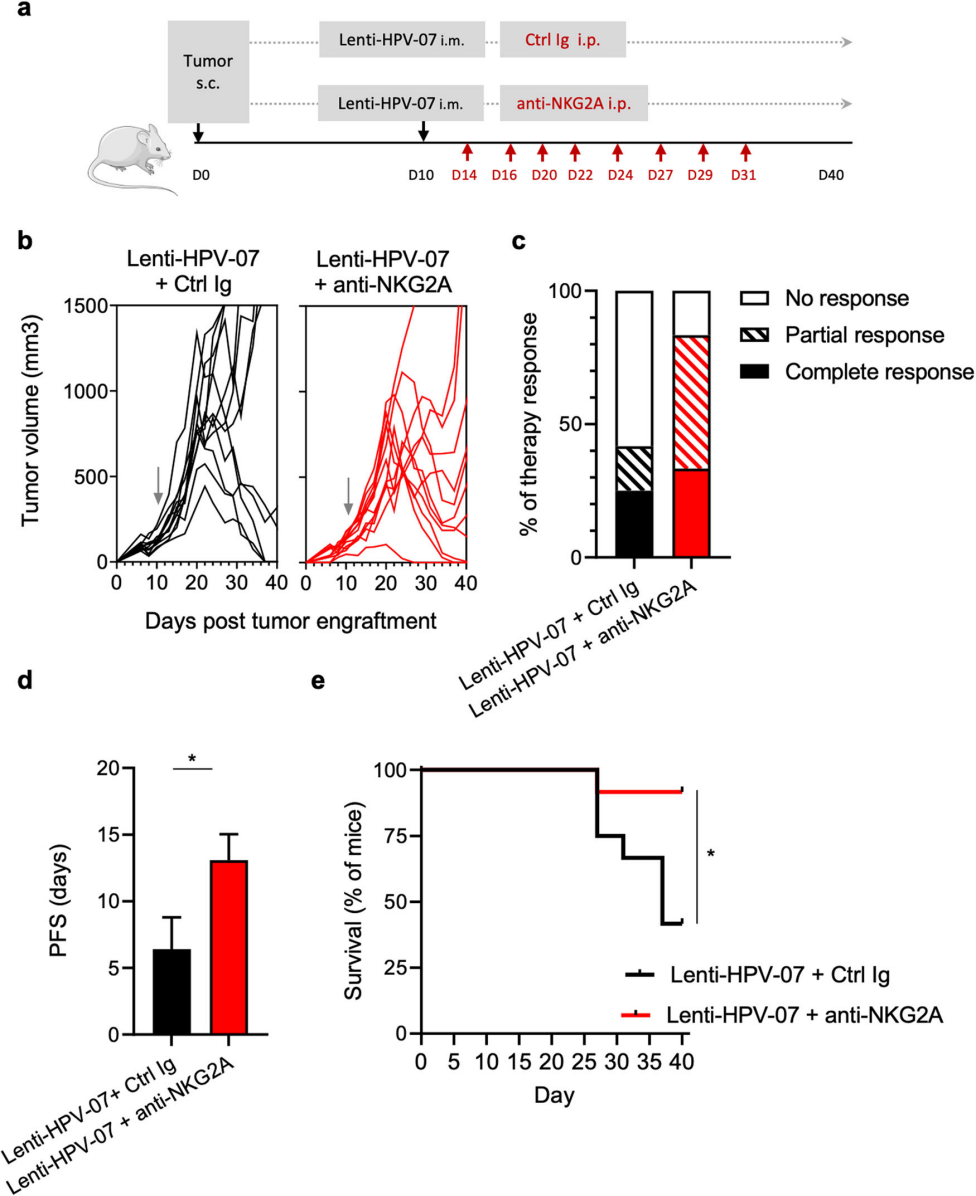
Increased efficacy of Lenti-HPV-07 and anti-NKG2A mAb in TC1 tumor regression. **a** Timeline of tumor engraftment and combinatory treatment with Lenti-HPV-07 and anti-NKG2A mAb. C57BL/6 mice (*n* = 12/group) were s.c. flank-engrafted with 1 × 10^6^ of TC1 cells. When the tumor volume reached an average of 120–140 mm^3^ (day 10), mice were randomized and vaccinated with the suboptimal dose of 1 × 10^8^ TU/mouse of Lenti-HPV-07. Mice were then treated 2-to-3 times a week with anti-NKG2A mAb (clone 20D5, Bioxcell) or Ig control (clone 2A3, Bioxcell). A total of 8 injections was given from day 14 to day 31 (200 µg/injection). **b** Spaghetti plots of tumor growth. **c** Therapy response rates according to RECIST criteria. **d** Progression-free survival time of mice bearing TC1 tumors treated with Lenti-HPV-07 and NKG2A blocking antibody. **e** Survival curves of the animals shown in (**b**). Statistical significance was determined using a Log-rank Mantel–Cox test, *p = 0.0134) in (**e**) or by unpaired *t*-test in (**d**). Mice were sacrificed when the size of the tumor volume reached 1500 mm^3^, in accordance with the defined humane endpoints. Error bars on the histograms represent standard deviation.

We then explored the effect of a suboptimal dose of Lenti-HPV-07 vaccination followed by anti-NKG2A therapy using 20D5 mAb in TC1 tumor-bearing C57BL/6 mice (Fig. 6a). We began the anti-NKG2A mAb treatment 4 days after Lenti-HPV-07 injection. Control animals received 1 × 10^8^ TU of Lenti-HPV-07 and a Ctrl Ig. Since it is well established that anti-NKG2A mAb treatment has no effect on TC1 tumor cell growth^28^, we did not include a group with anti-NKG2A mAb alone. In the Lenti-HPV-07 + Ctrl Ig group, only 5 of 12 mice achieved tumor regression the other 7 animals were unable to control tumor growth (Fig. 6b). In a striking contrast, 11 of 12 Lenti-HPV-07 + anti-NKG2A mAb mice achieved tumor regression while only 1 mouse was unable to control tumor growth (Fig. 6b). It is noteworthy that the removal of anti-NKG2A mAb treatment allowed tumor to relapse in 6 of 12 mice, strongly suggesting the need for repeated injections of anti-NKG2A to maintain the immune control of the tumor growth. Indeed, 80% of mice treated with Lenti-HPV-07 + anti-NKG2A mAb displayed a complete or partial regression response versus only 40% of mice treated with Lenti-HPV-07 alone (Fig. 6c). The progression-free survival (PFS) time doubled in mice that received the combination of Lenti-HPV-07 and anti-NKG2A mAb compared to the mice treated with the Lenti-HPV-07 + Ctrl Ig (Fig. 6d). Accordingly, the survival was significantly increased in the group with combinatory treatment (Fig. 6e). Because anti-NKG2A mAb alone had no impact on TC1 tumor growth^28^ and Lenti-HPV-07 + anti-NKG2A was more efficient than Lenti-HPV-07 + Ctrl Ig, the effect of combination therapy could be considered as synergistic rather than additive.

In summary, TIM3 or NKG2A blockade alone had no anti-tumor effect in our preclinical HPV-induced tumor model, probably due to the absence of TC1 tumor-induced T cells expected to be mobilized by ICI treatment. More importantly, these results show that a combination of lentiviral vector-based vaccination and ICI treatment, such as anti-TIM3 or anti-NKG2A mAb therapy, increases the therapeutic response.

## Discussion

We recently developed Lenti-HPV-07, a non-integrative, VSV-G-pseudotyped, immunotherapeutic lentiviral vector which induces robust and long-lasting T-cell responses against E6 and E7 HPV oncoproteins with various intensity according to the host MHC haplotype and HPV16 or 18 antigens^6^. Lenti-HPV-07 therapy is 100% effective in the preclinical murine model even on large HPV-induced tumors. Its mode of action is based on the induction of robust, resident memory and long-lasting CD8^+^ T cell-based immunity and deep remodeling of HPV-induced tumor immune microenvironment^6^. Even though in the preclinical model, a single i.m. injection of Lenti-HPV-07 is capable of full regression of solid tumors, a prime-boost regimen may be necessary to induce a sufficient anti-tumor T-cell immunity in clinical use. Moreover, it is often argued that prime-induced anti-vector immunity can weaken the efficacy of a homologous boost vaccination^11,12^. Our results clearly established that, despite the induction of anti-VSV-G_ind_ neutralizing antibodies and IFN-γ-producing T cells after a Lenti-HPV-07_ind_ prime, a homologous boost with Lenti-HPV-07_ind_ or a heterologous boost with Lenti-HPV-07_nj_ potentiated anti-HPV-07 T-cell effectors to a similar extent. It is possible that circulating anti-vector antibodies or systemic T cells do not come in contact soon, or efficiently enough, with the vaccinal pseudoviral particles injected in the muscle. Instead, these particles are rapidly taken up by local dendritic cells. Deemed to act upon the T-cell (re)activation, the dendritic cells might rest unaffected by prime-induced anti-vector immunity. This could also explain the absence of any negative effect of such immunity upon the homologous boost. Together, these results are important for the clinical introduction of Lenti-HPV-07 treatment suggesting unnecessary the separate production of GMP batches intended for prime and boost with distinct VSV-G pseudotyping. This considerably facilitates the production set-up and reduces the cost of vaccine for clinical trials.

We further performed a longitudinal NGS assay on peripheral blood lymphocytes to study the TCRβ and TCRα clonotype landscape throughout a Lenti-HPV-07 prime-boost protocol. The results demonstrated an evident expansion of certain TCRβ and TCRα clonotypes, paralleled by the increased frequencies of HPV-07-specific T cells post prime and a clonotype diversification post boost. Somewhat counter-intuitively, the increasing poly-clonality does not seem to favor the poly-specificity of the T-cell response in terms of the spectrum of recognized T-cell epitopes. As observed in our ELISPOT assay, T-cell response remained essentially directed against the E7_HPV16_ antigen both post prime and post boost in H-2^b^ mice. At the same time, no major response was detected post boost against the H-2^b^-restricted subdominant epitopes included in E6_HPV18_ and E7_HPV18_^6^. As a result, the boost would likely result into a more diverse TCR repertoire by the stimulation and expansion of novel low-affinity T-cell clones against the same epitopes. These clones have not been or have only been poorly stimulated post prime. As observed during yellow fever or SARS-CoV-2 vaccination in humans, the booster injection led to the expansion of rare specific clonotypes underrepresented post prime. These clonotypes demonstrated a lower reactivity to peptide stimulation suggesting a lower TCR affinity^29^.

The NGS-based TCR signature analysis failed to detect any significant difference in the clonotype panorama between mice boosted homologously with Lenti-HPV-07_ind_ and heterologously with Lenti-HPV-07_nj_. In both groups, T-cell clonotyping showed a preferential usage of TRBJ2.1, TRBJ2.7 Jβ segments and TRBV17 Vβ segment in the expanded clonotypes only in Lenti-HPV-07-immunized mice. Monitoring clonotypes specific to E7_HPV16_ RAHYNIVTF-epitope highlighted this preference and polarization of the TCRβ repertoire toward recognition of this immunodominant epitope, previously shown to be sufficient to induce T-cell mediated TC1 tumor eradication in C57BL/6 mice^6^.

Furthermore, the TCRα and TCRβ CDR3 amino acid sequence proximity evidenced some individual-specific clustering for each mouse, strongly suggesting that, after Lenti-HPV-07 immunization, each individual follows its own repertoire specialization. This observation is further interesting given that our syngeneic mice are likely endowed by a similar naive TCRαβ repertoire. Notably, distinctive CDR3 motifs associated with RAHYNIVTF-specific clonotypes were identified. Interestingly, these motifs were also detectable in a comparable study indicating that RAHYNIVTF-specific clonotypes are generated from a public TCR repertoire shared across syngeneic mice^30^.

In human, such divergence in the TCR repertoire specialization was demonstrated and explained by the large human leukocyte antigen (HLA) polymorphism. Moreover, an individual TCR repertoire specialization has been observed even in identical twins after yellow fever vaccination^31^. It is worth mentioning that a net perturbation of the TCRβ and TCRα was also detected in the Ctrl Lenti-injected mice. However, the Vα-/β- and Jα-/β-segment usage and the CDR3 sequences were clearly distinct between the Lenti-HPV-07- and Ctrl Lenti-injected groups. Therefore, a significant part of the TCRβ and TCRα repertoire perturbation may result from the immunity triggered against the vector backbone/adjuvant/formulation and not due to the global repertoire evolution from the baseline. Usually, the repertoire evolution is attributed to cognate TCRs specific to the target antigen^32,33^.

Overall, our results demonstrate the applicability of NGS to assay T-cell repertoire dynamics following i.m. immunization with Lenti-HPV-07. Peripheral blood cells could be used as sample. We further propose the use of NGS for in immune-monitoring of patients to be enrolled in the Lenti-HPV-07 Phase I/IIa clinical trial. However, co-detection of anti-vector T cell immunity could obscure the results. Therefore, use of NGS for immunomonitoring does not obviate the need for ELISPOT and intracellular cytokine staining to detect T-cell responses specific to the HPV-07 antigen encoded by the vaccine.

After the in-depth analysis of the Lenti-HPV-07-triggered T-cell response, we evaluated the anti-tumor effector functions of the induced T cells and the possibility of their unleashing by ICI treatments. We previously demonstrated that anti-PD1 treatment combined with suboptimal Lenti-HPV-07 vaccination synergize together to improve anti-tumor responses^6^. In the preclinical tumor model of HPV-induced cancer presented here, we combined Lenti-HPV-07 with TIM3 or NKG2A blockade ICI and demonstrated a significant increase in the frequencies of responding mice and their survival rate. Cell surface expression of TIM3 in T cells is triggered by the transcription factors T-bet and Nfil3 (Nuclear Factor, Interleukin 3 Regulated), IL-35 and other signals in the TME^34^. TIM3 has four ligands, i.e., carcinoembryonic antigen cell adhesion molecule 1 (CEACAM-1), high-mobility group protein B1 (HMGB1), and phosphatidylserine (PS), and Gal-9^18^. TIM3-Gal-9 interaction drives antigen-specific T cells into a state of exhaustion^21^. In fact, TIM3 expression characterizes the most terminally exhausted CD8^+^ T cells^22,34^. Stem-like CD8^+^ T cells, the main T-cell subset responsive to ICI, are characterized by TCF-1 expression. PD-1, but not TIM3, is frequently co-expressed with TCF-1^35^. Even though it has been postulated that TIM3 expression reduces the stemness of CD8^+^ T cells by antagonizing TCF-1^36^, our results clearly demonstrate that an anti-TIM3 antagonist treatment has a good unleashing potential toward the Lenti-HPV-07-induced effector T cells. Importantly, upregulation of TIM3 on T cells has been associated with the establishment of resistance to PD-1 blockade in cancer patients^37^. On the other hand, co-blockade of the TIM3 and PD-1 showed significant efficacy^22^. In addition to tumor infiltrating CD8^+^ T cells, dendritic cells also express TIM3 and thus anti-TIM3 antagonist treatment could act by regulating dendritic cell functions. In fact, TIM3 blockade activates NOD-like receptor family, pyrin domain containing 3 (NLRP3) inflammasome, resulting in the production of IL-1β and IL-18 effector cytokines by dendritic cells^38^. IL-18 induces IFN-γ production in tumor microenvironment and increases tumor immunogenicity^6^. Thus, there are multiple lines of support for anti-TIM3 therapy as an ICI to be either combined to anti-PD1 treatment or to replace it.

The C-type lectin NKG2A is an inhibitory receptor, mainly expressed by NK cells, but also by CD8^+^ T cells in tissue-resident and/or terminally exhausted CD8^+^ T cells in tumor context or upon chronic infection^39^. NKG2A is expressed as a heterodimer with another C-type lectin, CD94. NKG2A-CD94 complex interacts with the barely polymorphic non-classical MHC-I molecules, i.e., HLA-E and murine Qa-1^b^ ^29,30^. In contrast to classical MHC-I molecules, frequently lost by tumors, the expression of HLA-E is usually maintained by tumor cells^40^. NKG2A expression is induced by immunomodulators and/or TCR engagement^41^. NKG2A-CD94 receptor engagement delivers inhibitory signals to CD8^+^ T cells, dampening their effector activity, while blocking NKG2A unleashes the effector functions of NK and T cells. It is noteworthy that NKG2A is strongly expressed on resident memory T cells in HPV^+^ oropharyngeal cancers^42^. In TC1 tumor-bearing mice treated with Lenti-HPV-07, NK cells have no measurable anti-tumor effect, and the overall anti-tumor effect depends on CD8^+^ T cells readily mobilized by anti-NKG2A. In line with our observation, previous preclinical studies in TC1 tumor-bearing mice, showed that inhibition of NKG2A synergize successfully with therapeutic vaccination based on synthetic peptides. In this study, the anti-tumor effect of the therapeutic combination was relied on CD8^+^ T-cell effector functions, independently of NK cells^6,28^. Similarly, to anti-PD1 and anti-TIM-3, the combination of anti-PD-1 and anti-NKG2A treatment has beneficial anti-tumor effects^43^. In sum, we evidenced enhanced efficacy or synergistic effects of Lenti-HPV-07 vaccination with the combination treatment, irrespective of the chosen ICI. The results of the favorable effect of these alternative ICI on the anti-tumor effect of Lenti-HPV-07 may be instructive for future prospective Lenti-HPV-07 clinical trials.

Overall, our results validate the proof of concept for the use of anti-TIM3 or anti-NKG2A ICI when combined to Lenti-HPV-07 in further clinical trials, given that only 20–25% of cancer patients respond to ICI treatments alone.

Lenti-HPV-07 is entering a Phase I/IIa clinical trial for the immunotherapy of HPV16/18-related cervical or oropharyngeal cancers. The results generated here reiterate the potential benefits of the Lenti-HPV-07 immunotherapy and further elucidate its modes of action against malignant tumors caused by persistent HPV infections.

## Materials and methods

### Vector production and purification

All genes were *Homo sapiens* codon-optimized and synthetized by MWG eurofins then introduced in a pTRIP-B2M-Wm backbone by restriction digest and ligation between BamHI and XhoI sites^6^. Ctrl Lenti is a vector in which the transgene is replaced by a non-coding adapter. Lentiviral particles were produced by triple calcium phosphate transfection of HEK293T cells with (1) a plasmid (pTRIP) encoding vector RNA carrying the gene of interest, (2) a plasmid (pVSV-G) encoding VSV-G Indiana envelope protein, (3) a plasmid (pNDK D64V) encoding packaging proteins comprising an integrase-defective Pol protein (mutation D64V). Two days post-transfection, supernatants were harvested and clarified by centrifugation at 600 × *g* at 4 °C during 6 min. Lentiviral vectors were concentrated by ultracentrifugation at 90,000 × *g* during 1h30. Pellets were recovered in a buffer at pH 7.2 containing 25 mM PIPES (Sigma), 75 mM NaCl (Sigma) and 4% sucrose (Sigma). Lentiviral vectors were aliquoted and stored at −80 °C. Titration method has been described elsewhere^44^. The titer was defined as “Transduction Unit” (TU)/ml which is the number of LV particles per ml able to transfer its genetic material inside the reference HEK293T cells by transduction in presence of 8 µM of aphidicolin.

### Mice

Six- to 8-week-old female C57BL/6JRj mice supplied from Janvier (Le Genest Saint Isle, France) were used in this study. Mice were housed under standardized light–dark cycles in a temperature-controlled air-conditioned environment in ventilated cages under pathogen-free conditions at the Institut Pasteur animal facilities, Paris, France, with free access to food and water. All mouse studies were performed in accordance with the European and French guidelines (Directive 86/609/CEE and Decree 87-848 of 19 October 1987) after approval of the protocol by the Institut Pasteur Safety, Animal Care and Use Committee delivered by the local ethics committee (CETEA #DAP180049, CETEA #DAP190130 and the Ministry of High Education and Research APAFIS#16381-2018080217194542 v1, APAFIS# 20981-20190606164112731).

### Immunization

For immunogenicity studies, C57BL/6 awake mice were immunized intramuscularly with 1 × 10^9^ TU of Lenti-HPV-07 contained in 50 µl (Day 0 or Day 28). Mice primed at Day 0 were boosted with the same amount of Lenti-HPV-07 at Day 28. Splenocytes from all individual mice (*n* = 4/group) were studied at Day 42 (14 days post boost). At Days −2 before immunization, Days 12 and 26, and Day 42 (14 days post boost), 100–150 µl of blood were collected on awake animals by submandibular vein puncture into an Eppendorf tube containing 15 µl of heparin. Mice were monitored 2 times per week. Mice were euthanized by cervical dislocation according to predefined endpoints, i.e., at the end of the experimentation (day 42), or when animals exhibited signs of cachexia, prolonged behavioral abnormalities, or physical impairment.

### In vivo treatments

TC1 tumor cells (ATCC, CRL-2785) were cultured as previously described^6^. Subsequently, 1 × 10^6^ TC1 cells were subcutaneously injected into the flank of C57BL/6JRj mice under anesthesia initiated with isoflurane at a concentration of 2.5% and then maintained at 2% using a Compact Anesthesia Unit (Minerve). Ten to 13 days after tumor engraftment, awake mice were randomized and injected i.m. with a single injection of Lenti-HPV-07 or Ctrl Lenti diluted in 50 µl of PBS. Anti-TIM3 antibody (clone RMT3-23, BioXCell), anti-NKG2A antibody (clone 20D5, BioXCell) or Ig control (clone 2A3, BioXCell) were given 3–4 days after immunizations. Each antibody was administered i.p. 2 or 3 times per a week according to timeline shown in Figs. 5a and 6a (200 µg per mouse per injection) on awake animals. Monitoring of tumor growth was performed 2 or 3 times per a week with a digital caliper. Tumor volume was estimated as a sphere using the formula V = L × W × W/2, where V is the volume, L the length, and W the width. Mice were euthanized by cervical dislocation according to predefined endpoints, i.e., when tumor size reached 1500 mm^3^ or became ulcerated, or when animals exhibited signs of cachexia, prolonged behavioral abnormalities, or physical impairment.

### IFN-γ ELISPOT assay

As described previously^45^, T splenocyte responses were assessed by IFN-γ ELISPOT after in vitro stimulation with pools of peptides spanning the complete sequences of VSV-G_ind_ (Genscript) or each of the detoxified E6_HPV16_, E7_HPV16_, E6_HPV18_, or E7_HPV18_ antigens (Mimotopes) as encoded by Lenti-HPV-07. Splenocytes from individual immunized mice were homogenized, filtered through 70 µm-pore filters, and centrifuged for 5 min at 450 × *g*. Cells were then treated with Red Blood Cell Lysing Buffer (Sigma), washed twice in PBS and counted in a Chemometec (Nucleocounter NC-200) cell counter. Splenocytes were then plated at 1 × 10^5^ cells/well in 200 µl of RPMI-GlutaMAX, containing 10% heat-inactivated fetal calf serum, 100 U/ml penicillin and 100 mg/ml streptomycin, 1 × 10^–4^ M non-essential amino-acids, 1% vol/vol HEPES, 1 × 10^–3^ M sodium pyruvate and 5 × 10^–5^ M of β-mercaptoethanol in ELISPOT plates (Mouse IFN-γ ELISPOT^PLUS^, Mabtech). Cells were left unstimulated or were stimulated with pools of appropriate peptide pools at 2 µg/ml of each peptide) or with 5 µg/ml of Concanavalin A (Sigma) as a functionality control. For each mouse, the assay was performed in triplicate, according to the manufacturer’s recommendations. Plates were analyzed in an Immunospot CTL Immunospot S6 ultimate-V Analyzer (CTL). For the IFN-γ ELISPOT with a stimulation by lentiviral vectors, total proteins in concentrated lentiviral vector supernatants were quantified using the micro BCA Protein Assay kit (Thermo Scientific) following the manufacturer’s instructions.

### Neutralization assay

Neutralization assay was performed using serial dilutions of heat inactivated sera at 56 °C during 30 min, HEK293T cells and non-replicative LV particles pseudotyped with VSV-G_ind_ or VSV-G_nj_ which harbor the reporter luciferase firefly gene, to quantify host cell transduction. First, serial serum dilutions were incubated with 1.2 to 1.5 × 10^5^ TU of LV particles pseudotyped with VSV-G_ind_ or VSV-G_nj_ in clear flat-bottom 96-well black plates (Corning) during 30 min with gentle agitation at room temperature, in a final volume of 50 μl in DMEM-glutamax, completed with 10% heat-inactivated fetal calf serum and 50 U/ml penicillin and 50 U/ml streptomycin (Gibco). The samples were then mixed with 2 × 10^4^ HEK293T cells in 50 μl. After 3 days incubation at 37 °C 5% CO_2_, the transduction efficiency was determined by luminescence, using a Luciferase Assay System (Promega) on an EnSpire plate reader (PerkinElmer) using Enspire Manager v4.13 software.

### TCR sequencing

For bulk TCR RNAseq analysis, blood sample from retromandibular vein were recovered in a tube containing 10 µl of Heparin Choay (Cheplapharm). To recover peripherical blood cells from the blood samples, red cells were lysed using BD Pharm Lysis buffer (BD Biosciences) following the manufacturer’s instruction.

For the E7_HPV16_ RAHYNIVTF-epitope-specific TCR RNAseq analysis, splenocytes were isolated. CD8^+^ T cells were then enriched via negative selection using magnetic beads (Miltenyi) and then labeled through immune-staining utilizing antibodies including anti-CD8 (clone 53-6.7, Biolegend), anti-CD4 (clone RM4-5, eBioscience), anti-CD11b (clone M1/70, BD Biosciences), anti-B220 (clone RA3-6B2, eBioscience), and a RAHYNIVTF-specific H2-D^b^ dextramer (Immudex). The CD8^+^dextramer^+^cell population was then isolated via sorting using a BD FACSymphony™ S6 flow cytometer (BD Biosciences) and BD FACSChorus™ Software v1.1.

RNA was extracted from blood samples using RNeasy Mini Kit (Qiagen) per the manufacturer’s instructions. RNA quality was validated on a NanoDrop 2000c (Thermo Fisher Scientific) making sure that samples have the absorbance ratio 260/230 nm > 2.0, and further using the RNA 6000 Pico kit for 2100 Bioanalyzer (Agilent). The RNA integrity number was >6.5. TCRα and TCRβ DNA libraries were prepared simultaneously using SMARTer^®^ MouseTCR α/β Profiling Kit (Takara Bio).

Sequencing was performed on an Illumina Miseq using MiSeq Reagent Kit v3 (600-cycle) (Illumina) using MiSeq Software v4.1.0. About 1.5–3.0 million paired-end reads were generated per sample. Raw sequences assembly, quality control, and sequence annotation were performed on MiXCR v4.3.2 software using SMARTer Mouse TCRα/β Profiling Kit dedicated pipeline with a downsampling to homogenize samples^46^. Data analysis was performed on R using the Immunarch 0.9.1 R package^47^ and in-lab scripts. The CDR3 amino acid sequences were further analyzed using WebLogo software to generate sequence logos visualizing sequence conservation and variability^48^.

### Statistical analyses

Normal distribution of each group was tested using a Shapiro–Wilk test (*p* < 0.05) All two-way ANOVA were performed using a Geisser-Greenhouse correction. Statistical analysis was Prism GraphPad v9. In Figs. 2–5, data were represented as mean values and error bars represent SEM. Mann–Whitney unpaired *t*-test and Log-rank Mantel–Cox test were used to evaluate the statistical significance between groups.

### Reporting summary

Further information on research design is available in the Nature Research Reporting Summary linked to this article.

### Supplementary information

Supplemental Material

Reporting Summary

## Data Availability

The published article includes all datasets generated and analyzed during this study. All plasmids and lentiviral vectors generated in this study will be available under an MTA for research use, given a pending patent directed to Lenti-HPV-07 vaccination vectors. Further information and requests for resources and reagents should be directed to and will be fulfilled by the corresponding authors laleh.majlessi@pasteur.fr or francois.anna@pasteur.fr. TCR sequencing datasets generated during the current study are available in the Biostudies online repository under accession number: S-BSST1234.

## Code availability

In-lab scripts were used to generate chord diagram figures in R. Scripts are available on request by contacting corresponding author: francois.anna@pasteur.fr.
