# Supplementary material for: T-cell immunity induced and reshaped by an anti-HPV immuno-oncotherapeutic lentiviral vector
Source: NPJ Vaccines. 2024 Jun 10;9:102. doi: 10.1038/s41541-024-00894-0 (PMC11164992; doi:10.1038/s41541-024-00894-0)
Supplement: Supplementary file 1 — Supplemental Material [file 41541_2024_894_MOESM1_ESM.pdf]

T-cell immunity induced and reshaped by an Anti-HPV immuno-oncotherapeutic lentiviral vector

Ingrid Fert[1€], Laëtitia Douguet[1€], Benjamin Vesin[1€], Fanny Moncoq[1], Amandine Noirat[1], Pierre Authié[1], Sylvain Ciret[1], Fabien Le Chevalier[1], Catherine Blanc[1], Yakov Vitrenko[2], Pierre Charneau[1*$], Laleh Majlessi[1*$], and François Anna[1*$]

[1] Pasteur-TheraVectys Joint Lab, Institut Pasteur, Université de Paris, Virology Department, 28 rue du Dr. Roux, Paris F-75015, France

[2] Institut Pasteur, Université Paris Cité, Plate-forme Technologique Biomics, F-75015 Paris, France

[€] These authors contributed equally.

* Correspondence should be addressed to L.M. (laleh.majlessi@pasteur.fr) and F.A. (francois.anna@pasteur.fr)

[$] Senior authors

Supplemental Informations

Supplementary Table 1. Predicted VSV-G_{ind}-derived T-cell epitopes in H-2^b mice.

Predicted T-cell epitopes for H-2^b	a.a. sequence	Restricting element	Score*	a.a sequence identity**	Contained in the VSV-G pool #
VSV-G_{ind}:376-384	WAPY**E**DVE**I**	H-2D^b	19	5/9	5
VSV-G_{ind}:7-14	LAFL**F**IG**V**	H-2K^b	19	1/8	1
VSV-G_{ind}:14-21	VNCK**F**TI**V**		19	3/8	1
VSV-G_{ind}:394-401	SGYK**F**PL**Y**		19	6/8	5

*http://www.syfpeithi.de/bin/MHCServer.dll/EpitopePrediction.htm. This algorithm predicts the ligation strength to MHC molecules. The score is defined by the probability for an a.a. sequence of being processed and presented by MHC restricting elements. The score cut off of 19 has been chosen. The anchor residues are indicated in bold. ** a.a sequence identity between VSV-G_{ind} and VSV-G_{nj} inside the predicted epitope.

Supplementary Table 2. RAHYNIVTF-specific CD8$^+$ T splenocytes sorting summary.

Mouse	% of Dextramer$^+$ among CD8$^+$ T cells	Total number of purified cells
**P1**	5.2	110323
**P2**	3.6	93034
**P3**	6.5	263003
**P4**	6.9	351363
**PB1**	1.9	22042
**PB2**	5.6	188041
**PB3**	2.3	93859
**PB4**	2.8	93509

Table summarizing the sorting of RAHYNIVTF-specific CD8+ T splenocytes used in Fig. 4 and Supplementary Fig. 8. CD8+ enriched splenocytes from two groups of four mice, either mono-injected (Px) or prime-boosted (PBx), were sorted based on their labeling with a PE-conjugated H-2Db (RAHYNIVTF) dextramer. The table includes the percentage and total number of purified Dextramer+ cells among the parental CD8+ T cell population.

Supplementary Fig. 1. Alignement (Blastp) of glycoproteins G from Vesicular Stomatitis Virus (VSV-G) serotypes Indiana (ind) and New Jersey (nj).

```
ind  1     MKCLLYLAFLFIGVNCKFTIVFPHNQKGNWKNVPSNYHYCPSSSDLNWHNDLIGTALQVK  60
           M   L  A     +  K  IVFP +   G+WK VP  Y+YCP+S+D N H    G   +++
nj   1     MLSYLIFALAVSPILGKIEIVFPQHTTGDWKRVPHEYNYCPTSADKNSHGTQTGIPVELT  60

ind  61    MPKSHKAIQADGWMCHASKWVTTCDFRWYGPKYITHSIRSFTPSVEQCKESIEQTKQGTW  120
           MPK     Q +G+MCH++ W+TTCDFRWYGPKYITHSI +  P+  QC E+I+  K G
nj   61    MPKGLTTHQVEGFMCHSALWMTTCDFRWYGPKYITHSIHNEEPTDYQCLEAIKSYKDGVS  120

ind  121   LNPGFPPQSCGYATVTDAEAVIVQVTPHHVLVDEYTGEWVDSQFINGKCSNYICPTVHNS  180
            NPGFPPQSCGY TVTDAEA IV VTPH V VDEYTGEW+D  FI G+C   IC TVHNS
nj   121   FNPGFPPQSCGYGTVTDAEAHIVTVTPHSVKVDEYTGEWIDPHFIGGRCKGQICETVHNS  180

ind  181   TTWHSDYKVKGLCDSNLISMDITFFSEDGELSSLGKEGTGFRSNYFAYETGGKACKMQYC  240
           T W +    + +C      +   FFS+  E++S+G   TG RSNYF Y +    CKM +C
nj   181   TKWFTSSDGESVCSQLFTLVGGIFFSDSEEITSMGLPETGIRSNYFPYISTEGICKMPFC  240

ind  241   KHWGVRLPSGVWFEMADKDLFAAAR----FPECPEGSSISAPSQTSVDVSLIQDVERILD  296
           +  G +L + +WF++ D DL    R      +C   SSI  P + + D+SLI DVERILD
nj   241   RKQGYKLKNDLWFQIMDPDLDKTVRDLPHIKDCDLSSSIITPGEHATDISLISDVERILD  300

ind  297   YSLCQETWSKIRAGLPISPVDLSYLAPKNPGTGPAFTIINGTLKYFETRYIRVDIAAPIL  356
           Y+LCQ TWSKI +G PI+PVDLSYL PKNPG GP FTIING+L YF ++Y+RV++ +P++
nj   301   YALCQNTWSKIESGEPITPVDLSYLGPKNPGVGPVFTIINGSLHYFTSKYLRVELESPVI  360

ind  357   SRMVGMISGTTTERELWDDWAPYEDVEIGPNGVLRTSSGYKFPLYMIGHGMLDSDLHLSS  416
            RM G ++GT   R+LWD W P+ +VEIGPNGVL+T  GYKFPL++IG G +DSD+ +
nj   361   PRMEGKVAGTRIVRQLWDQWFPFGEVEIGPNGVLKTKQGYKFPLHIIGTGEVDSDIKMER  420

ind  417   KAQVFEHPHIQDAASQLPDD---ESLFFGDTGLSKNPIELVEGWFSSWKSSIASFF-FII  472
             + +EHPHI+ A + L  D   E L++GDTG+SKNP+ELVEGWFS W+SS+      II
nj   421   VVKHWEHPHIEAAQTFLKKDDTGEVLYYGDTGVSKNPVELVEGWFSGWRSSLMGVLAVII  480

ind  473   GLIIGLFLVLRVGIHLCIKLKHTKKRQIY-TDIEMNRLGK  511
           G +I +FL+  +G+      L   K+R IY +D+EM
nj   481   GFVILMFLIKLIGV--LSSLFRPKRRPIYKSDVEMAHFR  517
```

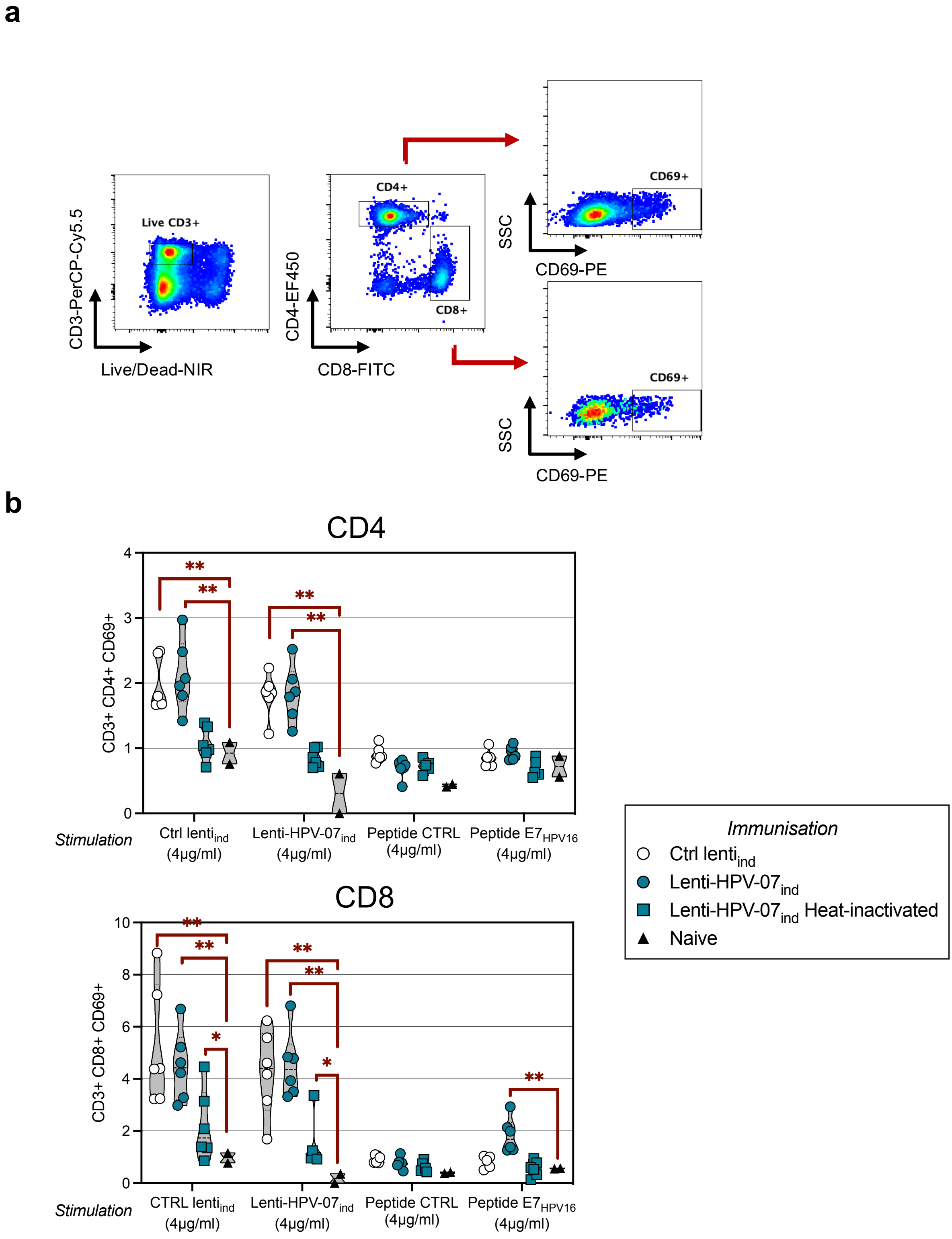
a
Live CD3+
CD3-PerCP-Cy5.5
Live/Dead-NIR
CD4+
CD8+
CD4-EF450
CD8-FITC
CD69+
SSC
CD69-PE
CD69+
SSC
CD69-PE
b
CD4
CD3+ CD4+ CD69+
**
**
**
**
Stimulation
Ctrl lenti_ind (4µg/ml)
Lenti-HPV-07_ind (4µg/ml)
Peptide CTRL (4µg/ml)
Peptide E7_HPV16 (4µg/ml)
CD8
CD3+ CD8+ CD69+
**
**
*
**
**
*
**
Stimulation
CTRL lenti_ind (4µg/ml)
Lenti-HPV-07_ind (4µg/ml)
Peptide CTRL (4µg/ml)
Peptide E7_HPV16 (4µg/ml)
Immunisation
Ctrl lenti_ind
Lenti-HPV-07_ind
Lenti-HPV-07_ind Heat-inactivated
Naive

Supplementary Fig. 2. Evaluation of non-specific splenocyte stimulation by Bone Marrow Derived Dendritic cells presentation of LV vaccines ex vivo.

Bone marrow cells from tibias and femur of naïves mice where cultured in complete RPMI 1640 (Gibco), 2mM L-glutamine (Gibco), 1% Penicillin /Streptomycin (Gibco), 10% FCS, 5Mm Hepes (Gibco), 50µM ß-mercapto-ethanol (Gibco), 1X non-essential amino acids (Gibco) and supplemented with 20 ng/ml of mouse GM-CSF during 5 days in Petri dishes. At day 3, fresh medium was added volume to volume. At day 5, cells were harvested with a scrapper and plated in 24-well plate at 2.8×10^5 per well and appropriate lentiviral vectors or peptides were added at a final concentration of 4 µg/ml. At day 6, splenocytes from mice vaccinated with Ctrl $lenti_{ind}$, Lenti-HPV-07_{ind}, heat-inactivated Lenti-$HPV7_{ind}$ ($n = 6$) or naïves ($n = 2$) were extracted and 5×10^6 cells were added per well in complete RPMI supplemented with agonist anti-CD28 (clone 37.51, BD Pharmigen) and anti-CD49b (clone 9C10, BD Pharmigen) antibodies at a final concentration of 1µg/ml. The splenocytes were recovered at D14 after the mono-injection or the boost injection of Lenti-HPV-07. At day 7 of the co-culture, splenocytes were recovered by pipetting, labelled by immunostaining with anti-CD3 (clone 17A2, Biolegend), anti-CD4 (clone RM4-5, eBioscience), anti-CD8 (clone 53-6.7, BD biosciences),anti-CD69 (clone H1.2F3, BD biosciences) and live/dead (invitrogen) and analyzed by flow cytometry (Attune Nxt, thermofisher). **a** Gating strategy. **b** Evaluation of early activation marker CD69 expression on $CD3^+$ $CD4^+$ or $CD3^+$ $CD8^+$ T splenocytes after ex vivo co-cultures with pulsed BM-DC. Statistical significance was determined using a Mann-Whitney test (*: $p < 0.05$; ** : $p < 0.01$).

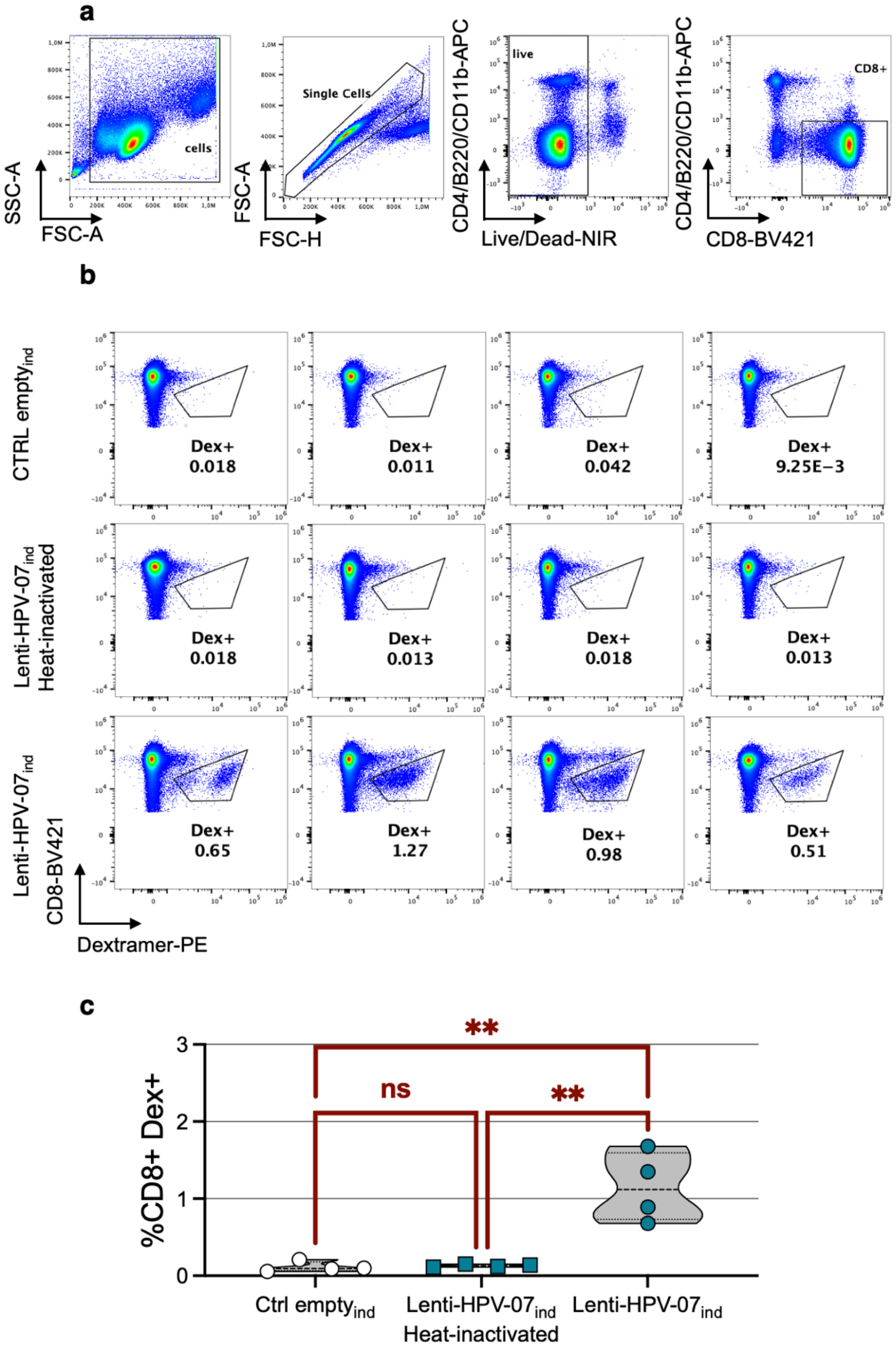

a
cells
SSC-A
FSC-A
Single Cells
FSC-A
FSC-H
live
CD4/B220/CD11b-APC
Live/Dead-NIR
CD8+
CD4/B220/CD11b-APC
CD8-BV421
b
CTRL empty$_{ind}$
Dex+ 0.018
Dex+ 0.011
Dex+ 0.042
Dex+ 9.25E−3
Lenti-HPV-07$_{ind}$ Heat-inactivated
Dex+ 0.018
Dex+ 0.013
Dex+ 0.018
Dex+ 0.013
Lenti-HPV-07$_{ind}$
Dex+ 0.65
Dex+ 1.27
Dex+ 0.98
Dex+ 0.51
CD8-BV421
Dextramer-PE
c
%CD8+ Dex+
**
ns
**
Ctrl empty$_{ind}$
Lenti-HPV-07$_{ind}$ Heat-inactivated
Lenti-HPV-07$_{ind}$

Supplementary Fig. 3 Tetramer-staining of $CD8^+$ T splenocytes specific to immunodominant RAHYNIVTF epitope-specific.

Splenocytes from mice injected with Ctrl $lenti_{ind}$, Lenti-$HPV7_{ind}$, or heat-inactivated Lenti-$HPV7_{ind}$ (n = 4) were extracted at D15 after the mono-injection or the boost injection and enriched in $CD8^+$ T cells by use of magnetic beads (Miltenyi) and stained with anti-CD3 (clone 17A2, Biolegend) anti-CD4 (clone RM4-5, eBioscience) anti-CD8 (clone 53-6.7 , BD biosciences), anti-B220 (clone RA3-602, BD biosciences), anti-CD11b (clone M1/70, BD biosciences), and live/dead (invitrogen) and analyzed by flow cytometry (Attune Nxt, thermofisher). CD4/CD11b/B220 labelling was used in parallel with CD8 labelling to gate out irrelevant cells **a** Gating strategy. **b** Dot plots of tetramer-staining in individual mice. **c** Recapitulative percentages of $CD8^+$ Tet^+ T cells in the experimental groups. Statistical significance was determined using a Mann-Whitney test (**: $p < 0.01$).

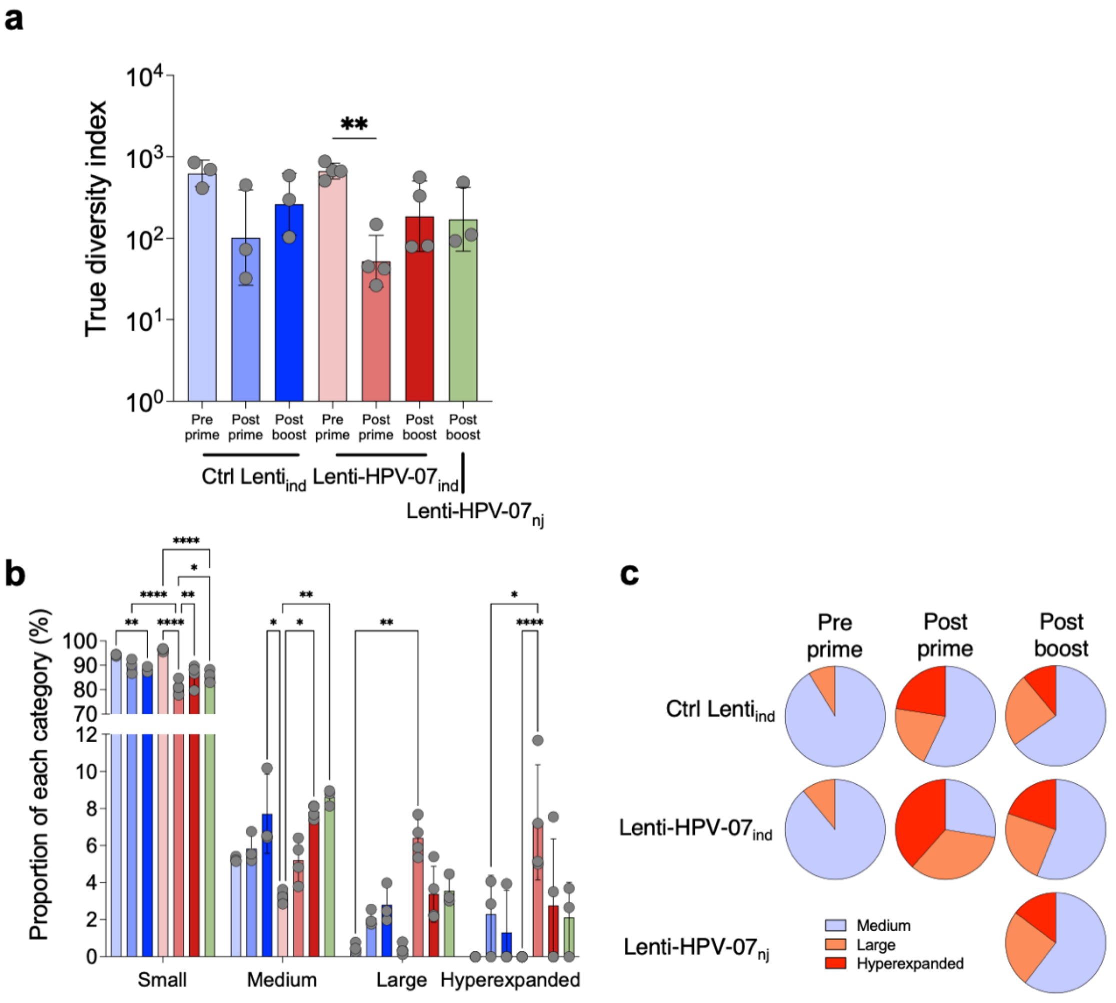

Supplementary Fig. 4. Dynamic evolution of TCRVα repertoire throughout a Lenti-HPV-07 prime-boost immunization regimen.

a Estimation of the TCRVα repertoire diversity using "true diversity" index. **d** Relative abundance of clonotypes, defined as their frequencies, compared to the total number of reads, which allows to divide the clonotypes into Small ($0 < X \leq 10^{-4}$), Medium ($10^{-4} < X \leq 10^{-3}$), Large ($10^{-3} < X \leq 10^{-2}$) and Hyperextended ($10^{-2} < X \leq 10^{0}$) categories. Relative abundance is the sum of the frequencies of all clonotypes from a given category. **c** Pie chart representing average (mean) proportions of Medium, Large and Hyperextended categories of each group. Statistical significance was determined using a one-way ANOVA in **(a)** and, Two-way ANOVA (* $p < 0.05$, ** $p < 0.01$, **** $p < 0.001$) in **(b)**. Error bars on the histograms represent standard deviation.

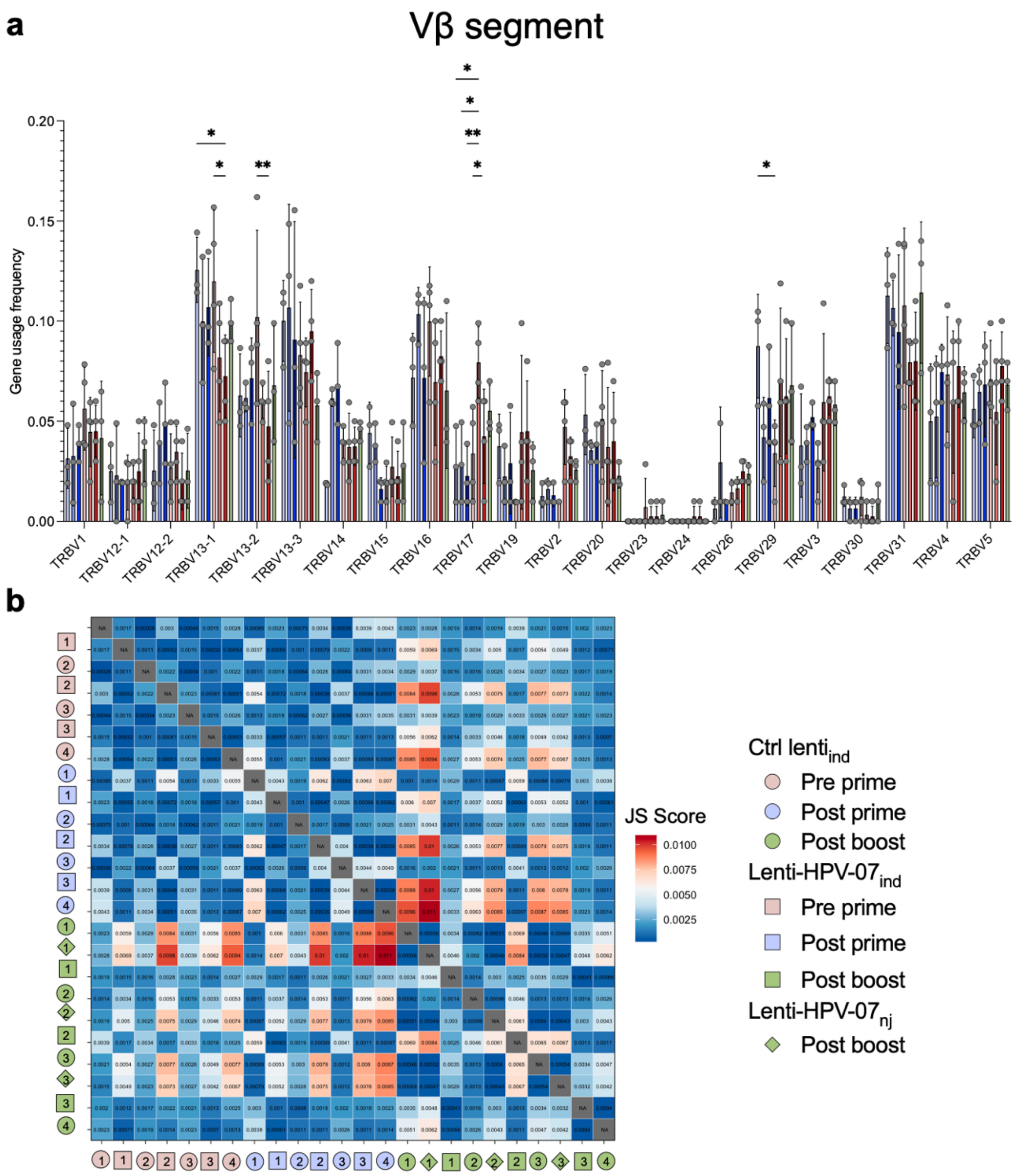

Supplementary Fig. 5. TCRVβ-gene segment usage in mice after various treatments.

a Frequencies of TCRVβ-gene segment usage in mice. Statistical significance was determined using a Two-wayANOVA (* $p < 0.05$, ** $p < 0.01$). **b** Correlation matrix of TCRVβ-gene segment usage among the mice, based on the Jensen-Shannon (JS) Divergence model. Error bars on the histograms represent standard deviation.

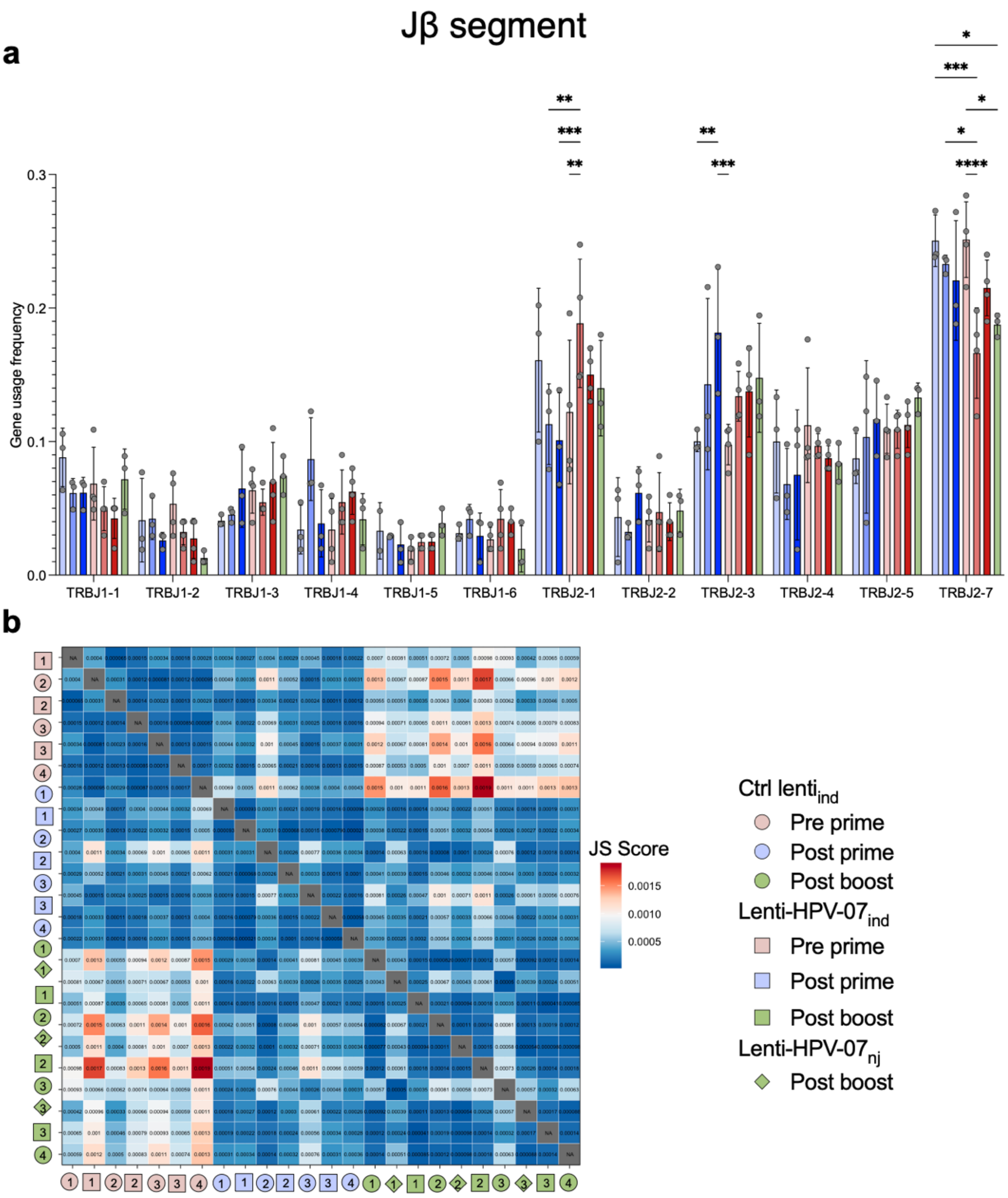

Supplementary Fig. 6. TCRJβ-gene segment usage in mice after various treatments

a Frequencies of TCRJβ-gene segment usage in mice. Statistical significance was determined using a Two-way ANOVA (* $p < 0.05$, ** $p < 0.01$). **b** Correlation matrix of TCRJβ-gene segment usage among the mice, based on Jensen-Shannon (JS) Divergence model. Error bars on the histograms represent standard deviation.

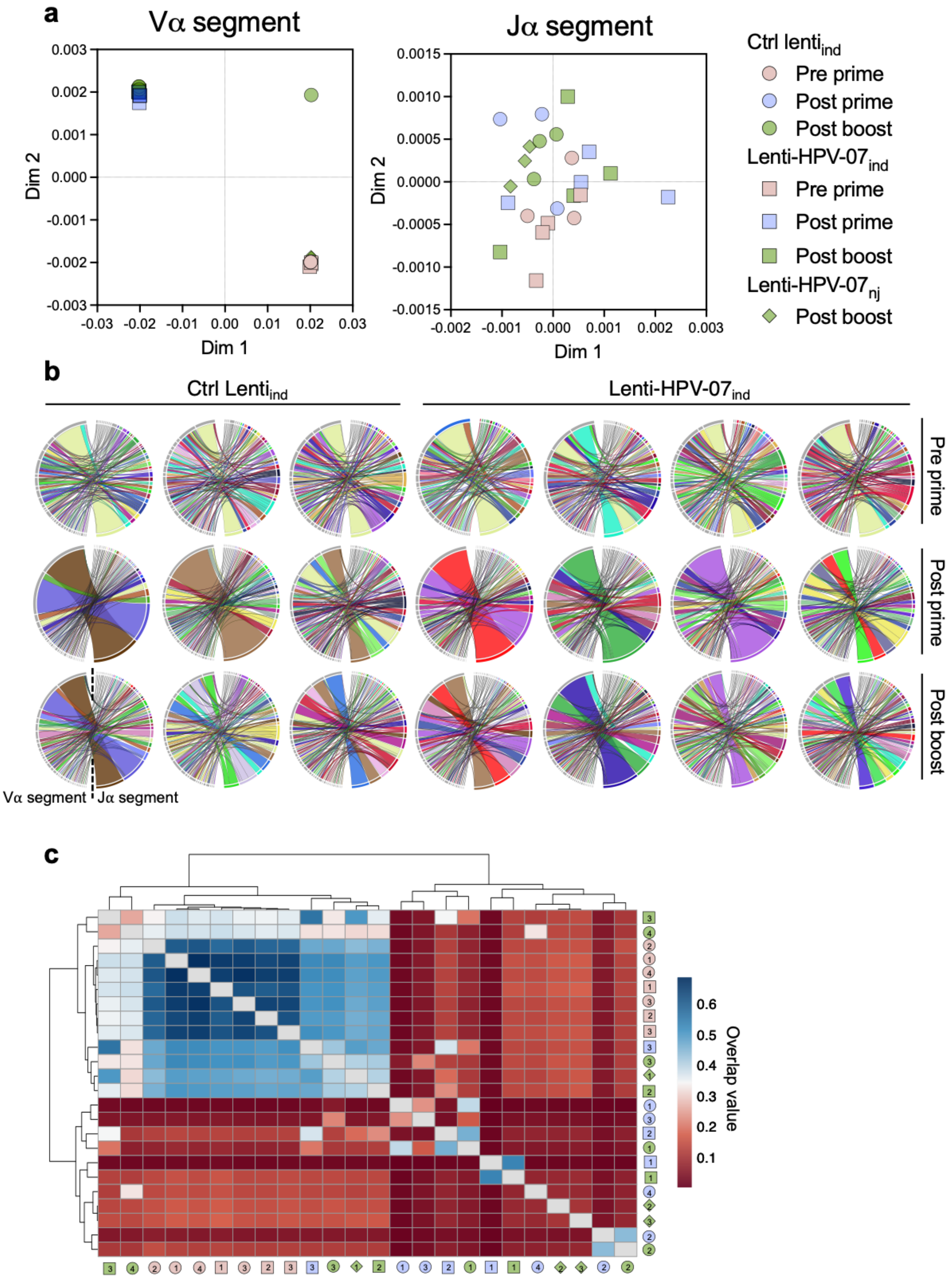
a
Vα segment
Jα segment
Dim 1
Dim 2
Ctrl lenti$_{ind}$
Pre prime
Post prime
Post boost
Lenti-HPV-07$_{ind}$
Pre prime
Post prime
Post boost
Lenti-HPV-07$_{nj}$
Post boost
b
Ctrl Lenti$_{ind}$
Lenti-HPV-07$_{ind}$
Pre prime
Post prime
Post boost
Vα segment
Jα segment
c
Overlap value

Supplementary Fig. 7. TCRVα-Jα rearrangements detected in Lenti-HPV-07-immunized mice.

Samples are those detailed in the Fig. 2**a** Jα- and Vα- gene segment usage in all clonotypes from each sample. For each clonotypes, Jα and Vα segments were identified, and the frequency of their usage was determined and are available in the Biostudies online repository under accession number: S-BSST1234. (https://www.ebi.ac.uk/biostudies/studies/S-BSST1234) Jα- and Vα-gene segment usage was compared among all mice at all timepoints according to a Jensen-Shannon Divergence model (available in the in the Biostudies online repository under accession number: S-BSST1234. (https://www.ebi.ac.uk/biostudies/studies/S-BSST1234) and treated by Multidimensional Scaling (MDS) to represent repertoire usage gene proximity on a 2-dimension plot. **b** Chord diagrams representing the Jβ and Vβ association in the top 100 most abundant clonotypes, as defined post boost. Chords are colored according to the Jα-gene segment.

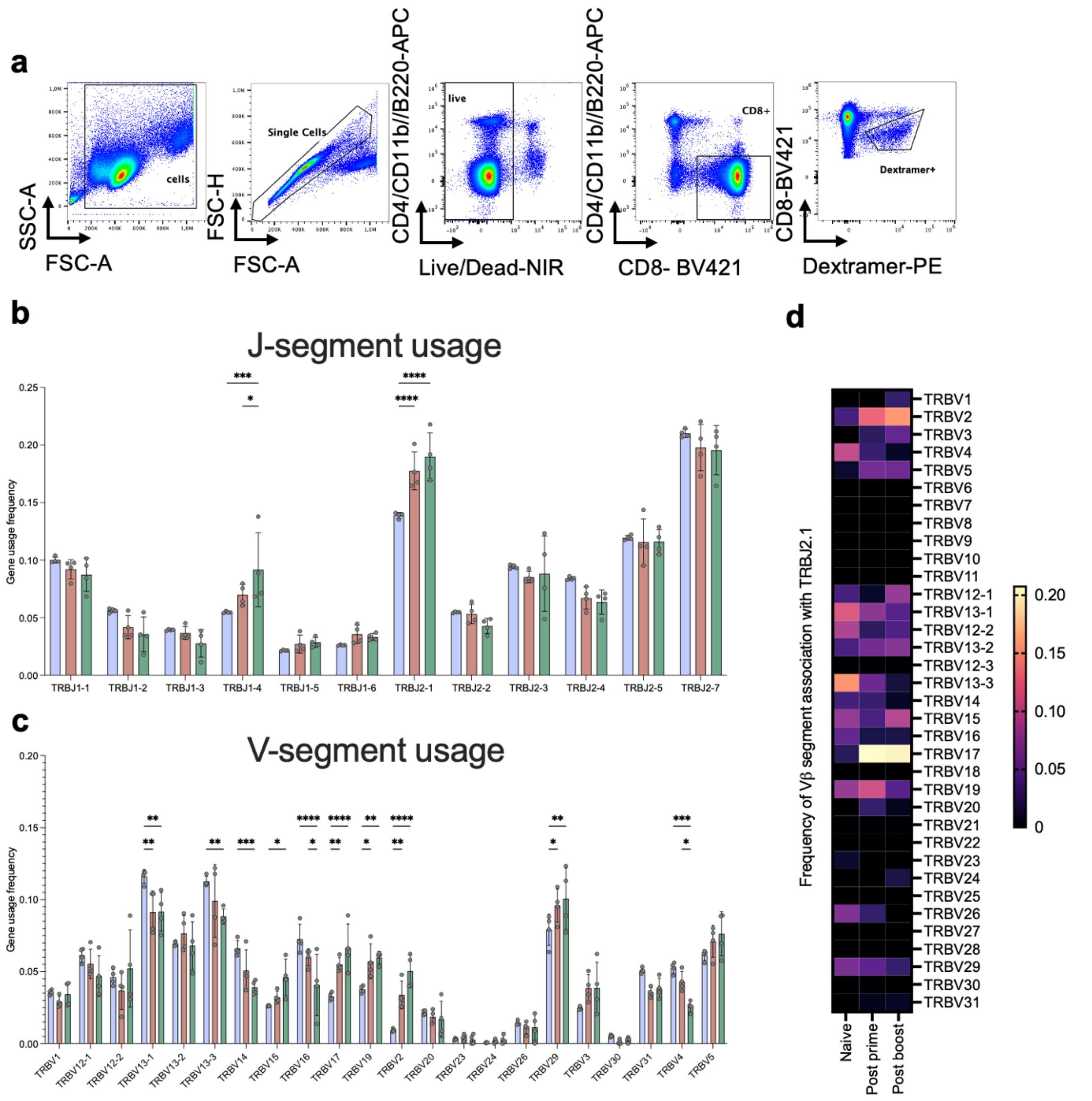

Supplementary Fig. 8. TCRVβ-Jβ rearrangements detected in in RAHYNIVTF-specific CD8^{+}T splenocytes in Lenti-HPV-07-immunized mice.

a Gating strategy of RAHYNIVTF-specific CD8^{+} T splenocytes for electronic sorting by flow cytometry. **b** Frequencies of TCRJβ gene segment usage in RAHYNIVTF-specific CD8^{+} clontoypes. **c** Frequencies of TCRVβ gene segment usage in RAHYNIVTF-specific CD8^{+} clontoypes. **d** Heatmap representing frequency of V-segment association with TRBJ2.1 J-segment in RAHYNIVTF-specific CD8^{+} clonotypes. Data are available in the in the Biostudies online repository under accession number: S-BSST1234. Statistical significance was determined using a Two-way ANOVA (* $p < 0.05$, ** $p < 0.01$). Error bars on the histograms represent standard deviation.
