# Supplementary material for: T-cell immunity induced and reshaped by an anti-HPV immuno-oncotherapeutic lentiviral vector
Source: NPJ Vaccines. 2024 Jun 10;9:102. doi: 10.1038/s41541-024-00894-0 (PMC11164992; doi:10.1038/s41541-024-00894-0)
Supplement: Supplementary file 2 — Reporting Summary [file 41541_2024_894_MOESM2_ESM.pdf]

Corresponding author(s): François ANNA
francois.anna@pasteur.fr

Last updated by author(s): 12/04/2024

Reporting Summary

Nature Portfolio wishes to improve the reproducibility of the work that we publish. This form provides structure for consistency and transparency in reporting. For further information on Nature Portfolio policies, see our Editorial Policies and the Editorial Policy Checklist.

Statistics

For all statistical analyses, confirm that the following items are present in the figure legend, table legend, main text, or Methods section.

n/a	Confirmed	
☐	☒	The exact sample size (*n*) for each experimental group/condition, given as a discrete number and unit of measurement
☐	☒	A statement on whether measurements were taken from distinct samples or whether the same sample was measured repeatedly
☐	☒	The statistical test(s) used AND whether they are one- or two-sided<br>*Only common tests should be described solely by name; describe more complex techniques in the Methods section.*
☐	☒	A description of all covariates tested
☐	☒	A description of any assumptions or corrections, such as tests of normality and adjustment for multiple comparisons
☐	☒	A full description of the statistical parameters including central tendency (e.g. means) or other basic estimates (e.g. regression coefficient) AND variation (e.g. standard deviation) or associated estimates of uncertainty (e.g. confidence intervals)
☒	☐	For null hypothesis testing, the test statistic (e.g. *F*, *t*, *r*) with confidence intervals, effect sizes, degrees of freedom and *P* value noted<br>*Give P values as exact values whenever suitable.*
☒	☐	For Bayesian analysis, information on the choice of priors and Markov chain Monte Carlo settings
☒	☐	For hierarchical and complex designs, identification of the appropriate level for tests and full reporting of outcomes
☒	☐	Estimates of effect sizes (e.g. Cohen's *d*, Pearson's *r*), indicating how they were calculated

Our web collection on statistics for biologists contains articles on many of the points above.

Software and code

Policy information about availability of computer code

Data collection	RNAseq was performed using Illumina MiSeq Software v4.1.0<br>ELISPOT counting was performed using Immunospot CTL Immunospot S6 ultimate-V Analyser v1.8<br>Cell sorting was performed using BD FACSChorus™ Software v1.1<br>BMDC assay flow cytometry was performed Attune NxT Software v 3.1<br>Neutralization assay was performed using Enspire Manager 4.13
Data analysis	For data analysis used software were:<br>Graphprism 10.1.0 for graph generation and statistical analysis<br>FlowJo 10.10.0 for Flow cytometry analysis<br>Mixcr 4.3.2 for RNAseq TCRbeta/alpha repertoire assembly and quality control<br>Immunarch R package 0.9.1 for TCRbeta/alpha repertoire analysis

For manuscripts utilizing custom algorithms or software that are central to the research but not yet described in published literature, software must be made available to editors and reviewers. We strongly encourage code deposition in a community repository (e.g. GitHub). See the Nature Portfolio guidelines for submitting code & software for further information.

Data

Policy information about availability of data

All manuscripts must include a data availability statement. This statement should provide the following information, where applicable:

- Accession codes, unique identifiers, or web links for publicly available datasets
- A description of any restrictions on data availability
- For clinical datasets or third party data, please ensure that the statement adheres to our policy

The published article includes all datasets generated and analyzed during this study. All plasmids and lentiviral vectors generated in this study will be available under an MTA for research use, given a pending patent directed to Lenti-HPV-07 vaccination vectors. Further information and requests for resources and reagents should be directed to and will be fulfilled by the corresponding authors laleh.majlessi@pasteur.fr or francois.anna@pasteur.fr. The datasets generated during the current study are available in the Biostudies online repository under accession number: S-BSST1234. (https://www.ebi.ac.uk/biostudies/studies/S-BSST1234)

Research involving human participants, their data, or biological material

Policy information about studies with human participants or human data. See also policy information about sex, gender (identity/presentation), and sexual orientation and race, ethnicity and racism.

Reporting on sex and gender	N/A
Reporting on race, ethnicity, or other socially relevant groupings	N/A
Population characteristics	N/A
Recruitment	N/A
Ethics oversight	N/A

Note that full information on the approval of the study protocol must also be provided in the manuscript.

Field-specific reporting

Please select the one below that is the best fit for your research. If you are not sure, read the appropriate sections before making your selection.

☒ Life sciences ☐ Behavioural & social sciences ☐ Ecological, evolutionary & environmental sciences

For a reference copy of the document with all sections, see nature.com/documents/nr-reporting-summary-flat.pdf

Life sciences study design

All studies must disclose on these points even when the disclosure is negative.

Sample size	Sample size were determined with the help of a bio-statistician. Previous datasets on similiar topics were used to determine size effect /group effects and optimal sample size to obtain statiscal significance on relevant compared groups.
Data exclusions	No data were excluded during this study
Replication	All experiments were performed only once but duplicate or triplicate in acquisition were always performed when possible (ELISPOT and Neutralization assay)
Randomization	Cage randomization with animals were performed to prevent cage effect. No further sample randomization was performed during experiments.
Blinding	The experiment operators were unaware of the specific group details in the study.

Behavioural & social sciences study design

All studies must disclose on these points even when the disclosure is negative.

Study description	N/A
Research sample	N/A

Sampling strategy	N/A
Data collection	N/A
Timing	N/A
Data exclusions	N/A
Non-participation	N/A
Randomization	N/A

Ecological, evolutionary & environmental sciences study design

All studies must disclose on these points even when the disclosure is negative.

Study description	N/A
Research sample	N/A
Sampling strategy	N/A
Data collection	N/A
Timing and spatial scale	N/A
Data exclusions	N/A
Reproducibility	N/A
Randomization	N/A
Blinding	N/A

Did the study involve field work? ☐ Yes ☐ No

Field work, collection and transport

Field conditions	N/A
Location	N/A
Access & import/export	N/A
Disturbance	N/A

Reporting for specific materials, systems and methods

We require information from authors about some types of materials, experimental systems and methods used in many studies. Here, indicate whether each material, system or method listed is relevant to your study. If you are not sure if a list item applies to your research, read the appropriate section before selecting a response.

Materials & experimental systems

n/a	Involved in the study
☐	☒ Antibodies
☐	☒ Eukaryotic cell lines
☒	☐ Palaeontology and archaeology
☐	☒ Animals and other organisms
☒	☐ Clinical data
☒	☐ Dual use research of concern
☒	☐ Plants

Methods

n/a	Involved in the study
☒	☐ ChIP-seq
☐	☒ Flow cytometry
☒	☐ MRI-based neuroimaging

Antibodies

Antibodies used

BMDC assay
anti-CD3 PerCP-Cy5.5 clone 17A2, Biolegend, Ref: 100217 Lot: B188933
anti-CD4 EF450 clone RM4-5, eBioscience, Ref: 48-0042-82, Lot: 1967921
anti-CD8 FITC clone 53-6.7, Millipore, Ref : MABF1542, Lot: VP1801242
anti-CD69 PE clone H1.2F3, BD biosciences, Ref: 555318, Lot : N.D
FCBlock clone 2.4G2 BD pharmigen Ref: 553142, Lot : 6148596

RAHYNIVTF--specific T Cell sorting
anti-CD8 FITC clone 53-6.7, Millipore, Ref : MABF1542, Lot: VP1801242
anti-CD4 EF450 clone RM4-5, eBioscience, Ref: 17-0042-82, Lot: E07040-1635
anti-CD11b APC clone M1/70, BD Biosciences,APC, Ref: 553312, Lot: 1047216
anti-B220 APC clone RA3-6B2, eBioscience, APC, Ref : 17-0452-82, Lot : E07151-1635
RAHYNIVTF-PE-specific H2-Db dextramer, Immudex, Ref: JA02195 PE 50, Lot : 210527KB4
FCBlock clone 2.4G2 BD pharmigen Ref: 553142, Lot : 6148596

anti-TIM3 neutralizing antibody clone RMT3-23, BioXCell, Ref: BE0115, Lot: N.D
anti-NKG2A neutralizing antibody clone 20D5, BioXCell Ref: BE0321 Lot : N.D
Ig control clone 2A3, BioXCell, Ref: BE0089 , Lot: 849323M2

Validation

Commercial antibodies were all certified after a quality control by the manufacturer and working concentration of fluorochrome-labelled antibodies were validated on splenocytes from naive C57BL6/J female mice in a immunostaining experiment followed by a flow cytometry analysis.

Eukaryotic cell lines

Policy information about cell lines and Sex and Gender in Research

Cell line source(s)	TC1 tumor cells (ATCC, CRL-2785)
Authentication	E6 and E7 proteins form Human Papillomavirus 16 expression was determined by Western blot.
Mycoplasma contamination	No Mycoplasma contamination was detected using MycoAlert™ PLUS Mycoplasma Detection Kit (Lonza)
Commonly misidentified lines (See ICLAC register)	N/A

Palaeontology and Archaeology

Specimen provenance	N/A
Specimen deposition	N/A
Dating methods	N/A

☐ Tick this box to confirm that the raw and calibrated dates are available in the paper or in Supplementary Information.

Ethics oversight	N/A

Note that full information on the approval of the study protocol must also be provided in the manuscript.

Animals and other research organisms

Policy information about studies involving animals; ARRIVE guidelines recommended for reporting animal research, and Sex and Gender in Research

Laboratory animals	Six- to 8-week-old female C57BL/6JRj mice supplied from Janvier (Le Genest Saint Isle, France) were used in this study. Mice were housed under standardized light–dark cycles in a temperature-controlled air-conditioned environment in ventilated cages under pathogen-free conditions at the Institut Pasteur animal facilities, Paris, France, with free access to food and water. All mouse studies were performed in accordance with the European and French guidelines (Directive 86/609/CEE and Decree 87-848 of 19 October 1987) after approval of the protocol by the Institut Pasteur Safety, Animal Care and Use Committee delivered by the local ethics committee (CETEA #DAP180049, CETEA #DAP190130 and the Ministry of High Education and Research APAFIS#16381-2018080217194542 v1, APAFIS# 20981-2019060616411273l).
Wild animals	N/A
Reporting on sex	N/A
Field-collected samples	N/A
Ethics oversight	N/A

Note that full information on the approval of the study protocol must also be provided in the manuscript.

Clinical data

Policy information about clinical studies

All manuscripts should comply with the ICMJE guidelines for publication of clinical research and a completed CONSORT checklist must be included with all submissions.

Clinical trial registration	N/A
Study protocol	N/A
Data collection	N/A
Outcomes	N/A

Dual use research of concern

Policy information about dual use research of concern

Hazards

Could the accidental, deliberate or reckless misuse of agents or technologies generated in the work, or the application of information presented in the manuscript, pose a threat to:

No	Yes	
☐	☐	Public health
☐	☐	National security
☐	☐	Crops and/or livestock
☐	☐	Ecosystems
☐	☐	Any other significant area

Experiments of concern

Does the work involve any of these experiments of concern:

No	Yes	
☐	☐	Demonstrate how to render a vaccine ineffective
☐	☐	Confer resistance to therapeutically useful antibiotics or antiviral agents
☐	☐	Enhance the virulence of a pathogen or render a nonpathogen virulent
☐	☐	Increase transmissibility of a pathogen
☐	☐	Alter the host range of a pathogen
☐	☐	Enable evasion of diagnostic/detection modalities
☐	☐	Enable the weaponization of a biological agent or toxin
☐	☐	Any other potentially harmful combination of experiments and agents

Plants

Seed stocks	N/A
Novel plant genotypes	N/A
Authentication	N/A

ChIP-seq

Data deposition

☐ Confirm that both raw and final processed data have been deposited in a public database such as GEO.

☐ Confirm that you have deposited or provided access to graph files (e.g. BED files) for the called peaks.

Data access links<br>*May remain private before publication.*	*For "Initial submission" or "Revised version" documents, provide reviewer access links. For your "Final submission" document, provide a link to the deposited data.*
Files in database submission	*Provide a list of all files available in the database submission.*
Genome browser session<br>(e.g. UCSC)	*Provide a link to an anonymized genome browser session for "Initial submission" and "Revised version" documents only, to enable peer review. Write "no longer applicable" for "Final submission" documents.*

Methodology

Replicates	*Describe the experimental replicates, specifying number, type and replicate agreement.*
Sequencing depth	*Describe the sequencing depth for each experiment, providing the total number of reads, uniquely mapped reads, length of reads and whether they were paired- or single-end.*
Antibodies	*Describe the antibodies used for the ChIP-seq experiments; as applicable, provide supplier name, catalog number, clone name, and lot number.*
Peak calling parameters	*Specify the command line program and parameters used for read mapping and peak calling, including the ChIP, control and index files used.*
Data quality	*Describe the methods used to ensure data quality in full detail, including how many peaks are at FDR 5% and above 5-fold enrichment.*
Software	*Describe the software used to collect and analyze the ChIP-seq data. For custom code that has been deposited into a community repository, provide accession details.*

Flow Cytometry

Plots

Confirm that:

☒ The axis labels state the marker and fluorochrome used (e.g. CD4-FITC).

☒ The axis scales are clearly visible. Include numbers along axes only for bottom left plot of group (a 'group' is an analysis of identical markers).

☒ All plots are contour plots with outliers or pseudocolor plots.

☒ A numerical value for number of cells or percentage (with statistics) is provided.

Methodology

Sample preparation	Splenocytes from individual immunized mice were homogenized, filtered through 70 µm-pore filters, and centrifuged for 5 minutes at 450 g. Cells were then treated with Red Blood Cell Lysing Buffer (Sigma), washed twice in PBS and counted in a Chemometec (Nucleocounter NC-200) cell counter.
Instrument	a BD FACSymphony™ S6 flow cytometer (BD Biosciences).
Software	Cell sorting was performed using BD FACSChorus™ Software v1.1<br>BMDC assay flow cytometry was performed Attune NxT Software v 3.1
Cell population abundance	Abundance of the relevant population were validated when population exceeded 1000 relevant events.<br>In the case of cell sorting a summary table is presented in Supplementary Table. 2
Gating strategy	Gating strategy are presented in Supplementary fig. 2,3 and 8

☒ Tick this box to confirm that a figure exemplifying the gating strategy is provided in the Supplementary Information.

Magnetic resonance imaging

Experimental design

Design type	*Indicate task or resting state; event-related or block design.*
Design specifications	*Specify the number of blocks, trials or experimental units per session and/or subject, and specify the length of each trial or block (if trials are blocked) and interval between trials.*
Behavioral performance measures	*State number and/or type of variables recorded (e.g. correct button press, response time) and what statistics were used to establish that the subjects were performing the task as expected (e.g. mean, range, and/or standard deviation across subjects).*

Acquisition

Imaging type(s)	*Specify: functional, structural, diffusion, perfusion.*
Field strength	*Specify in Tesla*
Sequence & imaging parameters	*Specify the pulse sequence type (gradient echo, spin echo, etc.), imaging type (EPI, spiral, etc.), field of view, matrix size, slice thickness, orientation and TE/TR/flip angle.*
Area of acquisition	*State whether a whole brain scan was used OR define the area of acquisition, describing how the region was determined.*

Diffusion MRI ☐ Used ☐ Not used

Preprocessing

Preprocessing software	*Provide detail on software version and revision number and on specific parameters (model/functions, brain extraction, segmentation, smoothing kernel size, etc.).*
Normalization	*If data were normalized/standardized, describe the approach(es): specify linear or non-linear and define image types used for transformation OR indicate that data were not normalized and explain rationale for lack of normalization.*
Normalization template	*Describe the template used for normalization/transformation, specifying subject space or group standardized space (e.g. original Talairach, MNI305, ICBM152) OR indicate that the data were not normalized.*
Noise and artifact removal	*Describe your procedure(s) for artifact and structured noise removal, specifying motion parameters, tissue signals and physiological signals (heart rate, respiration).*

Volume censoring | *Define your software and/or method and criteria for volume censoring, and state the extent of such censoring.*

Statistical modeling & inference

Model type and settings | *Specify type (mass univariate, multivariate, RSA, predictive, etc.) and describe essential details of the model at the first and second levels (e.g. fixed, random or mixed effects; drift or auto-correlation).*

Effect(s) tested | *Define precise effect in terms of the task or stimulus conditions instead of psychological concepts and indicate whether ANOVA or factorial designs were used.*

Specify type of analysis: ☐ Whole brain ☐ ROI-based ☐ Both

Statistic type for inference | *Specify voxel-wise or cluster-wise and report all relevant parameters for cluster-wise methods.*

(See Eklund et al. 2016)

Correction | *Describe the type of correction and how it is obtained for multiple comparisons (e.g. FWE, FDR, permutation or Monte Carlo).*

Models & analysis

n/a	Involved in the study
☐	☐ Functional and/or effective connectivity
☐	☐ Graph analysis
☐	☐ Multivariate modeling or predictive analysis

Functional and/or effective connectivity | *Report the measures of dependence used and the model details (e.g. Pearson correlation, partial correlation, mutual information).*

Graph analysis | *Report the dependent variable and connectivity measure, specifying weighted graph or binarized graph, subject- or group-level, and the global and/or node summaries used (e.g. clustering coefficient, efficiency, etc.).*

Multivariate modeling and predictive analysis | *Specify independent variables, features extraction and dimension reduction, model, training and evaluation metrics.*
